# Structural Characteristics of PON1 with Leu55Met and Gln192Arg Variants Influencing Oxidative-Stress-Related Diseases: An Integrated Molecular Modeling and Dynamics Study

**DOI:** 10.3390/medicina59122060

**Published:** 2023-11-22

**Authors:** Sudhan M., Janakiraman V., Sheikh F. Ahmad, Sabry M. Attia, Talha Bin Emran, Rajesh B. Patil, Shiek S. S. J. Ahmed

**Affiliations:** 1 Drug Discovery and Multi-Omics Laboratory, Faculty of Allied Health Sciences, Chettinad Hospital and Research Institute, Chettinad Academy of Research and Education, Kelambakkam 603103, Tamil Nadu, India; 2 Department of Pharmacology and Toxicology, College of Pharmacy, King Saud University, Riyadh 11451, Saudi Arabia; 3 Department of Pathology and Laboratory Medicine, Warren Alpert Medical School, Brown University, Providence, RI 02912, USA; 4 Legorreta Cancer Center, Brown University, Providence, RI 02912, USA; 5 Department of Pharmacy, Faculty of Allied Health Sciences, Daffodil International University, Dhaka 1207, Bangladesh; 6 Department of Pharmaceutical Chemistry, Sinhgad Technical Education Societys, Sinhgad College of Pharmacy, Vadgaon (BK), Pune 411041, Maharashtra, India

**Keywords:** paraoxonase, variant, oxidative stress, molecular docking, structural modeling, lactones, molecular dynamics simulation

## Abstract

*Background and Objectives*: PON1 is a multi-functional antioxidant protein that hydrolyzes a variety of endogenous and exogenous substrates in the human system. Growing evidence suggests that the Leu55Met and Gln192Arg substitutions alter PON1 activity and are linked with a variety of oxidative-stress-related diseases. *Materials and Methods:* We implemented structural modeling and molecular dynamics (MD) simulation along with essential dynamics of PON1 and molecular docking with their endogenous (n = 4) and exogenous (n = 6) substrates to gain insights into conformational changes and binding affinity in order to characterize the specific functional ramifications of PON1 variants. *Results:* The Leu55Met variation had a higher root mean square deviation (0.249 nm) than the wild type (0.216 nm) and Gln192Arg (0.202 nm), implying increased protein flexibility. Furthermore, the essential dynamics analysis confirms the structural change in PON1 with Leu55Met vs. Gln192Arg and wild type. Additionally, PON1 with Leu55Met causes local conformational alterations at the substrate binding site, leading to changes in binding affinity with their substrates. *Conclusions:* Our findings highlight the structural consequences of the variants, which would increase understanding of the role of PON1 in the pathogenesis of oxidative-stress-related diseases, as well as the management of endogenous and exogenous chemicals in the treatment of diseases.

## 1. Introduction

Paraoxonase (PON) is a multi-functional protein with antioxidant and antiatherogenic properties that catalyzes the hydrolysis of numerous endogenous and exogenous compounds in the human body [1]. Paraoxonase is widely expressed in the liver and metabolizes carbamates, organophosphates cyclic carbonates, unsaturated aliphatic esters, aromatic carboxylic acid esters, and lactones [2]. Few of these exogenous compounds have the potential to be active mutagens and carcinogens [3]. Notably, the PON family encodes three (*PON1*, *PON2*, and *PON3*) independent genes located adjacently on chromosome 7q21–22; each gene encodes a unique protein with approximately 65% similarity in amino acids [4,5]. Among the paraoxonase family, *PON1* is one of the isoforms that encodes a calcium-dependent esterase with 355 amino acids [6]. PON1 circulates in the blood and has the ability to improve cholesterol transport via high-density lipoprotein (HDL), which protects against oxidative damage [7]. Additionally, PON1 regulates the oxidation process of low-density lipoprotein (LDL), a crucial process in mitigating the development of oxidative stress [8]. Particularly, decreased PON1 activity has been linked to increased oxidative stress, which may affect the anti-inflammatory properties associated with HDL [9]. As a result, PON1 is considered a protective enzyme for several oxidative-stress-related diseases, including cancer [10], diabetes [11], infertility [12,13], coronary artery disease [14], and rheumatoid arthritis (RA) [15]. For instance, the elevated levels of reactive oxygen species (ROS) in diabetic conditions cause an increase in mitochondrial superoxide, which ultimately damages tissues [11]. In cancer, a significant increase in oxidative stress facilitates DNA mutations, genome instability, and cell proliferation [10]. Likewise, oxidative stress in rheumatoid arthritis hastens the inflammatory response, resulting in progressive joint degeneration and permanent disability [15]. Oxidative stress has been found to have adverse effects on sperm quality, oocyte fertilization, and miscarriages in cases of infertility [12,13]. Moreover, increased oxidative stress induces the secretion of inflammatory cytokines, which subsequently results in microvascular dysfunction and vessel damage [14]. In addition, PON1 is involved in a variety of biochemical processes, including innate immunity [16], the detoxification of reactive molecules [17], the bioactivation of pharmaceutical compounds [18], endoplasmic reticulum stress modulation [19], and the regulation of cell proliferation and apoptosis [20].

Recent studies have demonstrated that genetic variations in the *PON1* gene cause significant effects on its enzyme activity [21,22,23]. For instance, the PON1 variant with glutamine at position 192 increases the protein’s ability to prevent the breakdown of oxidized lipids and shield LDL from oxidative changes [24]. In contrast, the presence of the arginine variant at position 192 contributes to elevate the levels of LDL, triglycerides, and total cholesterol, thereby increasing susceptibility to coronary artery disease [25]. In 2023, Alshammary et al. [26] revealed a correlation between the PON1 Q192R polymorphism and female infertility in Saudi women. Likewise, Fekih et al. (2017) demonstrated an association between PON1 L55M and Q192R variants and the development of diabetic nephropathy in individuals with type 1 diabetes [27]. In addition, PON1 variants were assessed for their association with age-related macular degeneration [28], colorectal cancer [29], lymphocytic leukemia [30], psoriasis [31], and amyotrophic lateral sclerosis [32]. 

**Figure 1:**
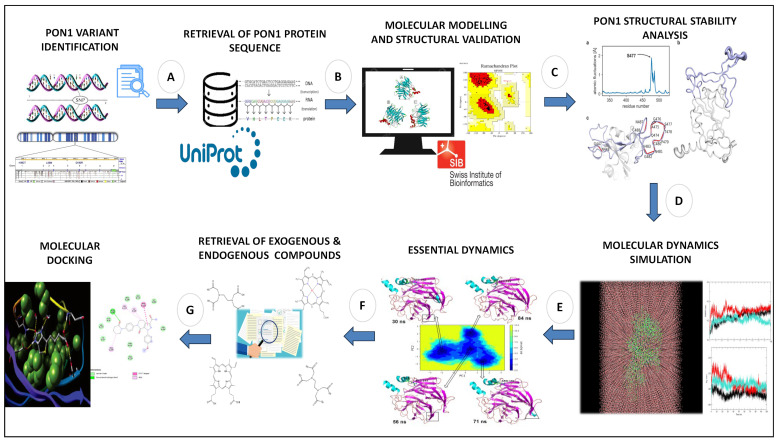
Complete workflow of the current investigation: (**A**) search for reported PON1 variant in literature databases, (**B**) retrieval of PON1 protein sequence, (**C**) molecular modelling and structural validation, (**D**) molecular dynamics simulation, (**E**) essential dynamics analysis, (**F**) retrieval of exogenous and endogenous substrates, and (**G**) molecular docking analysis.

Although Leu55Met and Gln192Arg variants have been linked to a number of diseases [28,29,30,31,32], it is still unknown how these amino acid substitutions affect the structural stability and substrate binding pockets of the PON1 protein. Therefore, this study aimed to implement a series of computational methods to evaluate the effects of Leu55Met and Gln192Arg substitutions on the PON1 structure stability and substrate binding affinity of its endogenous and exogenous molecules. Our overall workflow (Figure 1) includes (1) the generation and quality assessment of wild-type and variant (Leu55Met and Gln192Arg) PON1 structures; (2) protein stability assessment influenced by these variants; (3) molecular dynamics (MD) simulation with essential dynamics; and (4) molecular docking with their exogenous and endogenous substrates. Herein, our investigation shows the conformational changes in the PON1 structure in the presence of these variants contributing to destabilization and altered binding affinity of its exogenous and endogenous substances compared to wild-type PON1.

## 2. Materials and Methods

### 2.1. Retrieval of the PON1 Protein Sequence

Using accession number P27169, the amino acid sequence of human PON1 was obtained from the UniProt database (https://www.uniprot.org/, accessed on 30 April 2023). Simultaneously, the reported PON1 missense variants were searched in the literature databases (PUBMED and Google Scholar) utilizing a rational search with a keyword such as “human” or “Homo sapiens” in conjunction with a gene symbol (“*PON1*”) or protein name (“paraoxonase1”). The most frequently reported variants were chosen and incorporated into the wild-type protein sequence. Thereby, the three distinct sequences (seq1: wild-type, seq2: Leu55Met, and seq3: Gln192Arg) were generated and subjected to BLASTp analysis (Supplementary File S1).

### 2.2. Generation of Structures through Molecular Modelling

Keeping protein data bank (PDB) as a reference database, each PON1 sequence was subjected to NCBI’s BLASTp tool to search for the PON1 protein structure. Due to the lack of complete PON1 structure in the PDB database, we used the SWISS-MODEL web server (https://swissmodel.expasy.org/, accessed on 10 May 2023) to construct the three-dimensional homology model with the appropriate experimental structure as a template from PDB. SWISS-MODEL web server provides multiple structure templates to model PON1 protein structure. However, the optimal template structure was chosen using the qualitative model energy analysis (QMEAN) score, sequence coverage and identity, oligo state (monomer), global QMEANDisCo scores, and X-ray crystallography structure resolution [33]. Based on the selected template, the wild-type PON1 structure was generated and its quality was evaluated using the QMEAN, ProSa, VERIFY-3D, PROCHECK, and ERRAT tools [34]. Further, the variant structures were generated from the wild-type model by incorporating the amino acid changes (Leu55Met or Gln192Arg) using the VMD mutator plug-in version 1.9.4A53 (https://www.ks.uiuc.edu/Research/vmd/plugins/mutator/, accessed on 11 May 2023).

### 2.3. Evaluation of the Stability of PON1 Structures

Seven distinct protein stability prediction tools, such as MAESTRO Web, CUPSAT, DynaMut, PremPS, SDM2, I-Mutant 2.0, and HOPE, were employed to determine the structural stability and functional consequences of amino acid substitution [35,36,37,38,39,40,41]. All of these tools use distinct algorithm to assess the protein stability. For instance, MAESTRO Web analyzes the variant structure to calculate di-sulfide linkage and variant sensitivity profiles [35]. Similarly, CUPSAT assesses structural stability by calculating torsion potential and amino acid distance [36]. DynaMut predicts structural dynamics based on variations in the entropy of vibration [37]. The PremPS tool compared structural and evolutionary characteristics with the same number of stabilizing and destabilizing mutations in a symmetrical dataset [38]. Similarly, SDM2 generates a likelihood table for amino acid substitution by comparing the SNP structure to the specified homologous families [39]. I-Mutant uses support vector machine to identify and evaluate the effect of amino acid substitution [40]. The HOPE tool predicts the structural changes caused by the variant by comparing them to those of the protein in its wild state [41]. In addition, HOPE reveals differences in protein size, charge, hydrophobicity, and amino acid interactions between wild-type and mutated state of the analyzed protein. 

### 2.4. Preparation and Execution of MD Simulation

Three independent MD simulation systems were generated to assess the structural stability of PON1 proteins (A. wild-type, B. Leu55Met, and C. Gln192Arg). The GROMACS 2019 software was used to build the simulation system [42] with the Charmm36 force field [43]. PON1 structure was placed in a dodecahedron box with distances from each edge. Then, the TIP3P water model was used to solvate the dodecahedron box, and sodium ions (17 Na^+^ for the Gln192Arg; 18 Na^+^ for the wild type and Leu55Met) were added to maintain system neutrality [44]. The constant temperature (300 K), pressure (1 atm), and number of atoms are maintained by the Berendsen NVT ensemble and the NPT (constant number of particles, pressure, and temperature) ensembles [45]. Particle mesh Ewald and LINCS were used to maintain the electrostatic interaction (cut-off of 1.0 nm) and constrain bonds [46]. The protein structure was energy-minimized using the steepest descent approach for 50,000 steps. Finally, the MD simulation was conducted for 100 ns for each protein structure. Further, root mean squared fluctuation (RMSF), root mean squared deviation (RMSD), average solvent accessible surface area (SASA), the radius of gyration (Rg), and hydrogen bond (HB) trajectory analyses were carried out and plotted with the help of the XMgrace software version 5.1.25 [47]. Further, through the essential dynamics investigation, two principal components (PC1 and PC2) were obtained as eigen vectors, signifying the dominant motions in the respective protein [48]. These principal components were further used as the reaction coordinates in Gibb’s free energy surface analysis, and the lowest energy meta-stable conformations for each protein were identified. The effects of mutated residues on other residues of the protein were analyzed through residue–residue contacts and the contact frequency using the mdciao tool [49]. Further, the residue–residue dynamic cross-correlations were assessed through the dynamical cross-correlation matrix (DCCM) to determine how mutations influence the inner dynamics of protein conformations [50]. The covariance matrix was constructed for the Cα atoms of the systems, and the cross-correlations between atoms were computed to obtain the DCCM time-dependent plots. The DCCM values were between −1 and +1, presenting positive and negative correlation, respectively, in the DCCM plots.

### 2.5. Molecular Docking of PON1 with the Endogenous and Exogenous Substrates

The Glide module in Schrödinger software (Schrödinger Release 2018-2: Glide, Schrödinger, LLC, New York, NY, USA, 2018) was used to determine the differences in binding affinity of endogenous and exogenous substrate with wild-type and the variant PON1 structures. Four endogenous natural lactones, dihydrocoumarin, 2-hydroxy-gamma-butyrolactone, alpha-angelica lactone, gamma-nonalactone, and six exogenous oxidized unsaturated acids and synthetic molecules such as (2S, 3R) -3-amino-2-hydroxyheptonicacid, arachidonic acid, 4-hydroxy docosahexaenoic acid, dihydropyran, mevalonic acid, and 2-hydroxyvaleric acid involved in catalyzing the lactonization of PON1 were downloaded in SDF format from the PubChem database (https://pubchem.ncbi.nlm.nih.gov, accessed on 30 April 2023). To the collected structures, OPLS force field was applied using the LigPrep module in Maestro Version 11.2, Schrödinger. On the other hand, the protein prep module in Maestro (Schrödinger Release 2018-2, Schrödinger, LLC, New York, NY, USA, 2018) was used to optimize the PON1 structures, and then receptor grid generation module in Maestro Version 11.2, Schrödinger was used to create the grid around the substrate binding sites (HIS 115, HIS 134, ASN 168, PHE 222, ASN 224, ASN 270, ILE 291, and PHE 292), as reported by Tavori et al., 2010 [51]. Finally, the docking was performed for these ten substrates with PON1 wild-type and variant structures, and binding affinities were compared.

## 3. Results

### 3.1. Protein Modeling Validation of PON1 Structure

**Figure 2:**
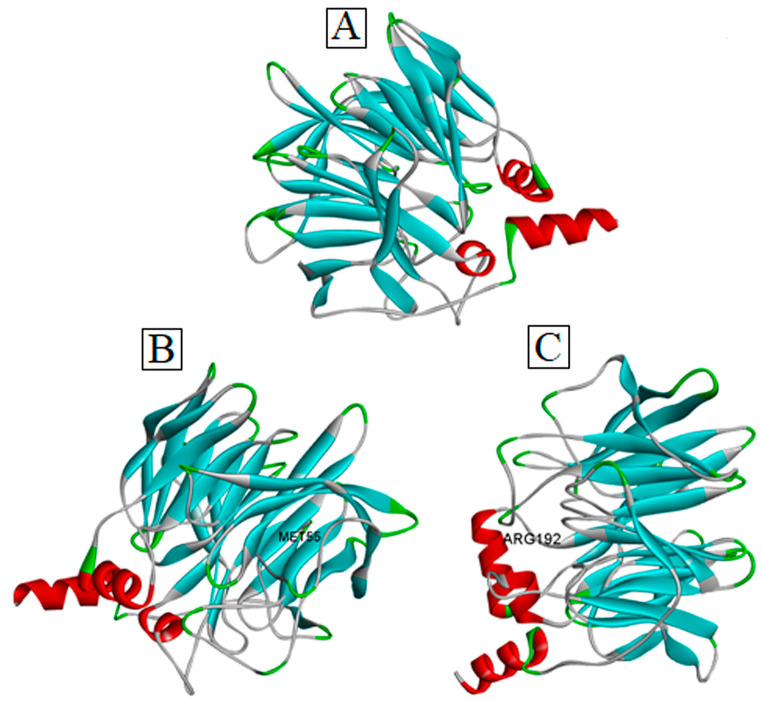
Three-dimensional structure of PON1 (**A**) wild-type, (**B**) Leu55Met, and (**C**) Gln192Arg.

Our extensive literature database search revealed that Leu55Met and Gln192Arg are the most frequently reported missense variants of PON1 associated with a wide range of diseases [28,29,30,31,32]. In accordance with the methodology, three distinct protein sequences were generated to search for the experimental protein structure of PON1 through the BLASTp tool in the protein data bank. Due to the unavailability of the experimentally derived PON1 structure, the wild-type structure (Figure 2) was modeled using a SWISS-MODEL server with the default parameters. Overall, ~50 templates were identified for the purpose of homology modelling through the SWISS-MODEL server, among which 6H0A was selected as a template to model the wild-type PON1 protein structure. The choice of selecting PDB ID: 6H0A was deemed superior after considering criteria such as the QMEAN score (0.93), sequence identity (82.25%), oligomeric state (monomer), QMEANDisCo global scores (0.90 ± 0.05), sequence coverage (95.2%), and X-ray crystallography structure resolution (2.10 Å) in comparison to other templates. Using the selected template, the three-dimensional protein structure of PON1 was generated, and its quality was determined using QMEAN, ProSa, VERIFY-3D, PROCHECK, and ERRAT. Based on the quality assessment, the modeled PON1 wild-type protein structure showed to have -8.07 (ProSa), 0.9 (QMEAN), 86.40% (PROCHECK), 90.56% (VERIFY3D), and 93% (ERRAT). Thus, our assessment confirmed that the generated PON1wild-type structure was suitable for generating Leu55Met and Gln192Arg variant structures, respectively (Figure 2).

### 3.2. Stability Analysis of Protein with Leu55Met and Gln192Arg Variant

The stability assessment with seven different tools showed that variant structures have a significant impact on protein stability (Table 1). Notably, the Leu55Met and Gln192Arg variants in the PON1 predicted to have destabilizing effects were outputted by most of the tools (MAESTRO Web, CUPSAT, I-Mutant, PremPS, and SDM2). In contrast, the DynaMut tool showed both variants as having stabilizing effects on the PON1 structure. On the other hand, HOPE predicts changes in protein properties due to the variant (Leu55Met and Gln192Arg). For instance, substitution of Leu55Met increases the size and causes more hydrophobic behavior than the wild type. Similarly, changes in Gln192Arg residues enhance positive charge and increase the protein size, which predictably affects protein folding and its function.

### 3.3. Effects of Leu55Met and Gln192Arg in Protein Dynamics 

**Figure 3:**
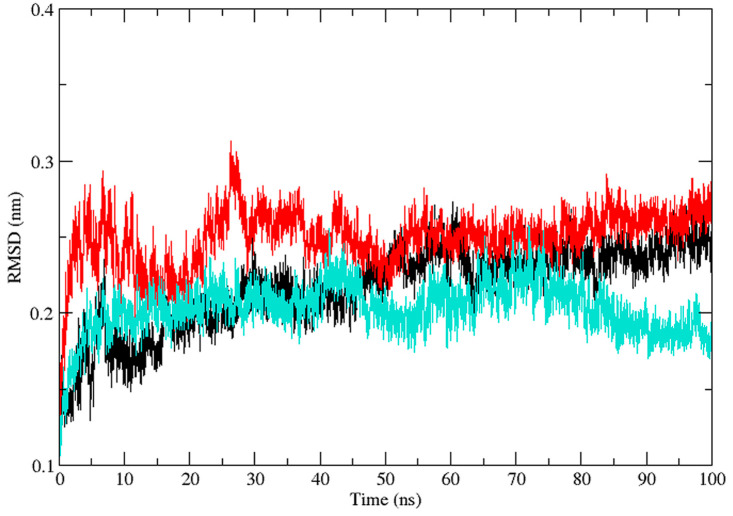
The plot of root mean square deviation (RMSD) (Backbone) values of PON1 wild-type (Black), Leu55Met (Red), and Gln192Arg (Cyan).

**Figure 4:**
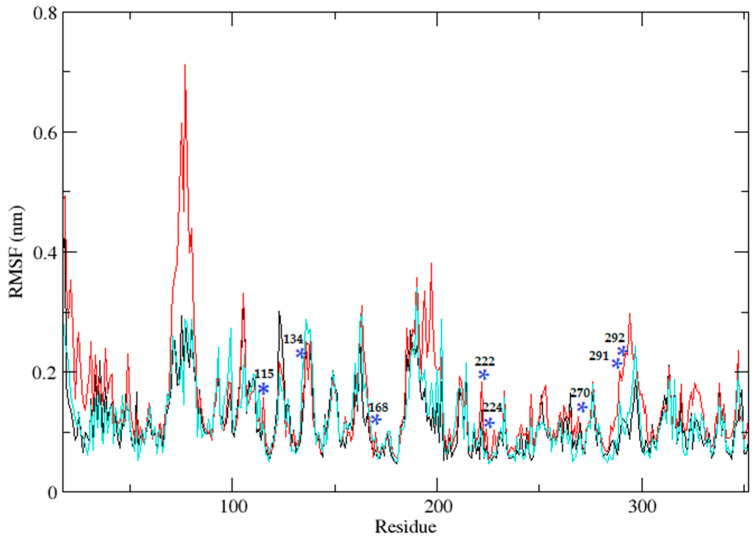
The plot illustrates the root mean square fluctuations (RMSF) of protein residues throughout a 100 ns molecular dynamics simulation for PON1 wild-type (black), as well as the Leu55Met (red) and the Gln192Arg (cyan). Blue * indicate the fluctuations at the substrate binding sites.

**Figure 5:**
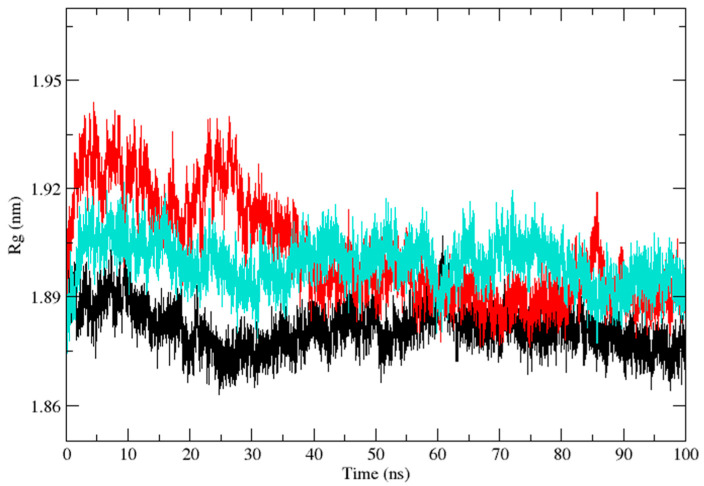
The plot of radiation of gyration (Rg) (Backbone) shows the compactness of the PON1 wild type (Black), Leu55Met (Red), and Gln192Arg (Cyan).

**Figure 6:**
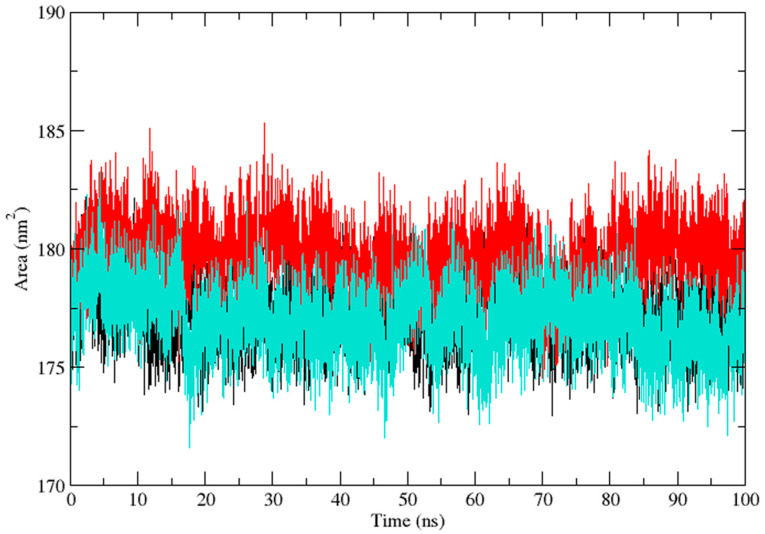
The plot of solvent accessible surface (SASA) (Protein) area demonstrates the PON1 wild type (Black), Leu55Met (Red), and Gln192Arg (Cyan) values during the 100 ns molecular dynamics simulation.

**Figure 7:**
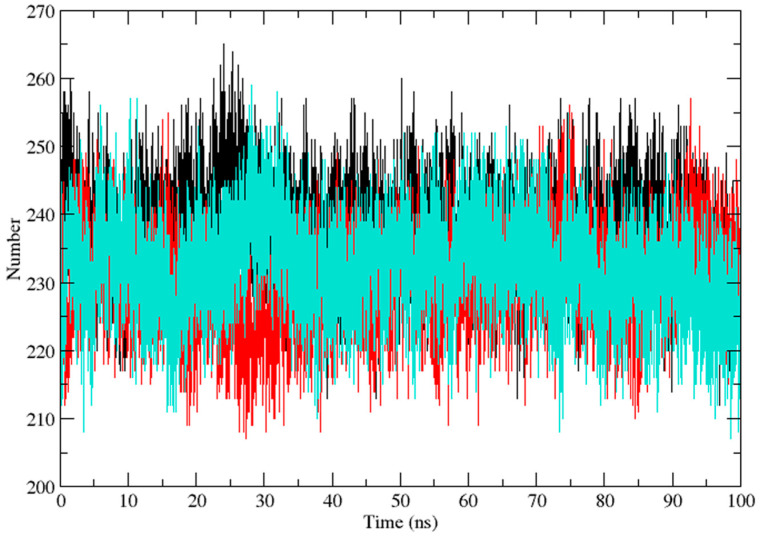
The plot of hydrogen bond (HB) (Backbone) shows the intramolecular hydrogen number of PON1 wild-type (Black), Leu55Met (Red), and Gln192Arg (Cyan).

Three independent MD simulations were performed to understand the stability of (A) wild-type, (B) Leu55Met, and (C) Gln192Arg PON1 structures. The MD simulation had a run time of 100 ns and produced RMSD, RMSF, Rg, SASA, and HB trajectories for each protein. These trajectories demonstrate the crucial dynamic behavior of the protein. For instance, if the average RMSD presents less than 0.30 nm, it represents a stable conformation of protein [52,53]. Figure 3 shows the RMSD trajectory of PON1 wild-type and variants (Leu55Met and Gln192Arg). Herein, the average RMSD values of wild-type (0.216 nm), Leu55Met (0.249 nm), and Gln192Arg (0.202 nm) confirm the lower stability of Leu55Met than the wild-type and Gln192Arg PON1 protein. Similarly, RMSF indicated the fluctuation of the protein residues during the simulation; the average RMSF being less than 0.3 nm denotes less protein flexibility [54]. In our case, the RMSF (Figure 4) of the Leu55Met variant (0.150 nm) showed high fluctuation at certain protein residues compared to the wild-type (0.118 nm) and Gln192Arg (0.120 nm) PON1. Additionally, the RMSF plot (Figure 4) showed fluctuation at the substrate binding sites, which may alter the binding affinities of the substrates in variants compared to wild type. Next, the Rg determines the protein’s structural compactness; a lower Rg value indicates a more compact structure [54]. The PON1 wild type and variants’ Rg results are depicted in Figure 5. The average Rg value of the PON1 wild type was 1.88 nm, Leu55Met was 1.90 nm, and Gln192Arg was 1.89 nm. The SASA demonstrates the surface area of the protein’s interaction with the solvent environment [54]. Figure 6 shows the SASA plots of the wild type, Leu55Met, and Gln192Arg. The average SASA of the PON1 wild type, Leu55Met, and Gln192Arg were 177.34 nm^2^, 179.71 nm^2^, and 177.00 nm^2^, respectively. Similarly, the number of hydrogen bonds formed in the wild type and variants is depicted in Figure 7. The average HB of the PON1 wild type, Leu55Met, and Gln192Arg were 238, 230, and 232, respectively. The HB attributes affect the intermolecular interactions within the protein. 

#### 3.3.1. Essential Dynamics of Leu55Met PON1

**Figure 8:**
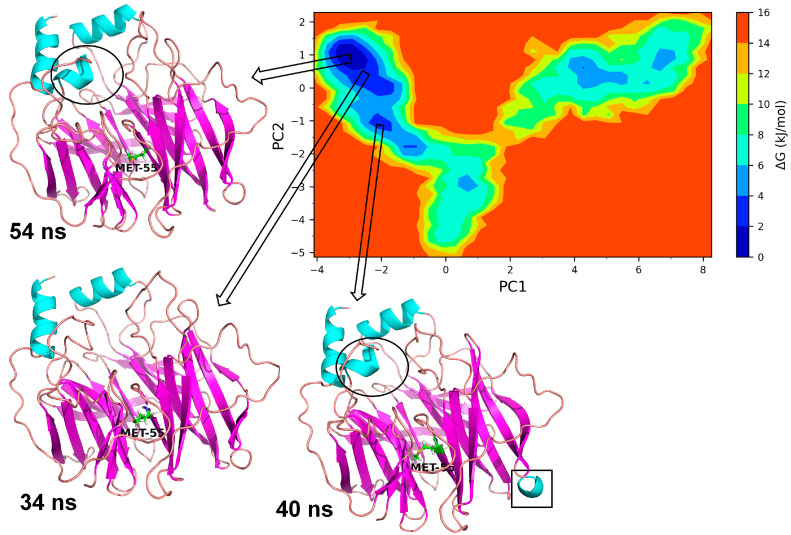
Gibb’s free energy analysis for Leu55Met PON1. The α-helix made up of residues Gly288 to Asp295 is shown in circles; while the α-helix made up of Gln147 to Lys151 is shown in square box.

**Figure 9:**
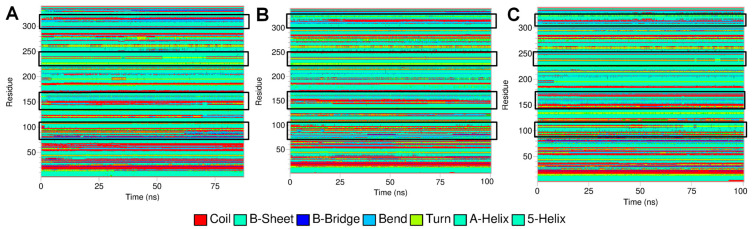
Secondary structural changes obtained from DSSP analysis. (**A**) Leu55Met PON1, (**B**) Gln192Arg PON1, and (**C**) wild-type PON1.

The essential dynamics were performed and two principal components (PC1 and PC2) were obtained for the MD trajectory set of each protein. These principal components were used as the reactions coordinates in the Gibb’s free energy surface (FES) analysis. The large positive values on Gibb’s FES plots represent the highly correlated conformations, while the high negative values represent the anti-correlated conformations. The energy basin with dark blue color shows the region with lowest energy meta-stable conformations, while lighter shades of the blue region show the slightly higher energy conformations with some conformational barriers. In the case of Leu55MetPON1, a small energy basin between −2.8 and −3.2 on PC1 and 0.8 and 1.2 on PC2 showed the lowest energy conformations (Figure 8). The representative lowest energy conformation from 54 ns showed three distinct α-helices. Another energy basin surrounding this lowest energy basin showed slightly higher energy conformations, where the conformational barrier was observed to be in the loop region with residues Gly288 to Asp295, where a secondary structural change to the α-helix resulted in the meta-stable conformation. A small energy basin around −2 on PC1 and around −1 on PC2 also showed three distinct α-helices along with a small α-helix in residues Gln147 to Lys151. The secondary structural changes are shown in Figure 9. Overall, the low energy conformations were fewer than the higher energy conformations for Leu55Met PON1.

#### 3.3.2. Essential Dynamics of Gly192Arg PON1

**Figure 10:**
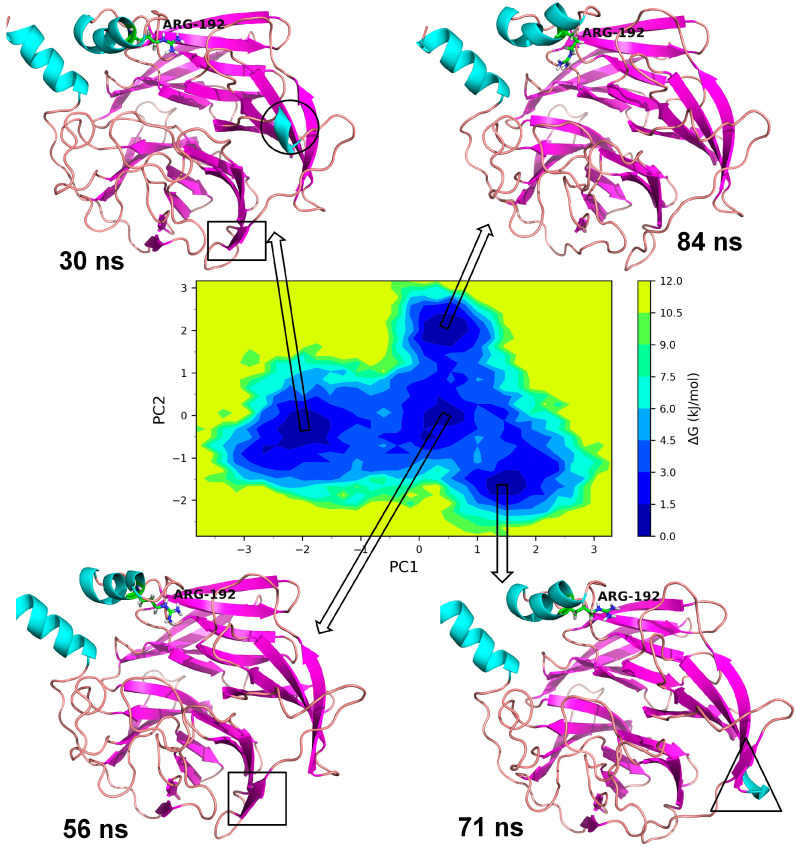
Gibb’s free energy analysis for Gln192Arg PON1. The α-helix made up of residues Val109 to Ser111 is shown in circles, the β-sheet made up of Cys42 to Val45 is shown in square box, and α-helix made up of Gln147 to Lys151 is shown in triangle.

**Figure 11:**
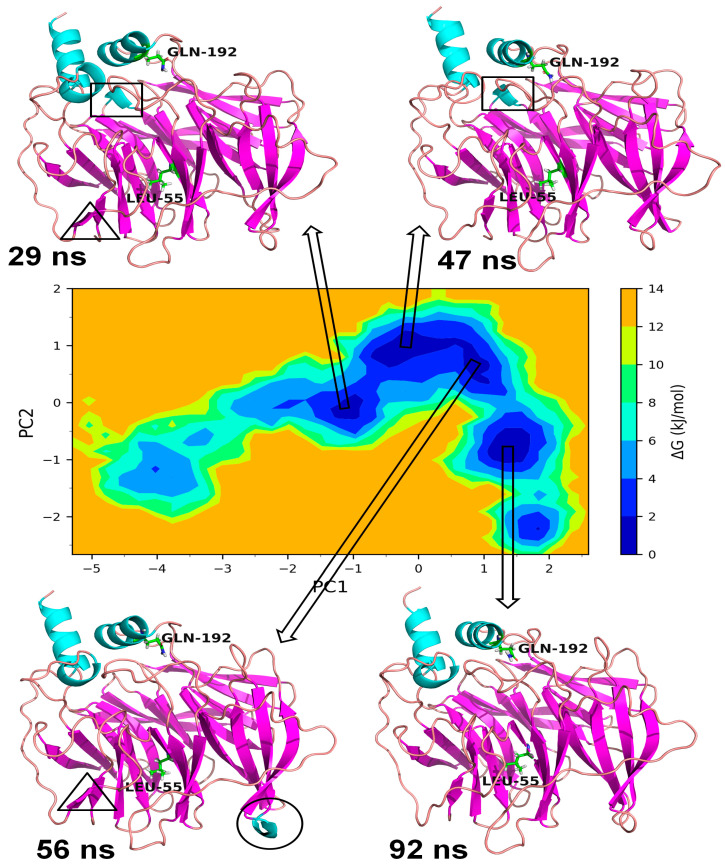
Gibb’s free energy analysis for wild-type PON1. The α-helix made up of residues Gly288 to Asp195 is shown in square box, the β-sheet made up of Cys42 to Val45 is shown in triangle, and α-helix made up of Gln147 to Lys151 is shown in a circle.

In the case of Gly192Arg PON1, there were four distinct lowest energy basins: one in anti-correlating region with high negative values on both PCs, one in the correlating region with high positive values on both PCs, one in the non-correlating region with values around zero on both PCs, and one in the anti-correlating region with high negative values on PC2. The largest numbers of conformations were found in the energy basin centered around −2 on PC1 and −0.5 on PC2 (Figure 10). The representative conformation, i.e., the trajectory at 30 ns, showed three distinct α-helices. The small α-helix that occurred from secondary structural change in the loop region residues Val109 to Ser111 was unique in this lowest energy conformation. A small β-sheet originating from the residues Cys42 to Val45 was also unique in this lowest energy conformation. Both of these unique secondary structures were not found in the lowest energy conformations in the second energy basin occupying 0 to 0.5 on PC1 and 1.5 to 2.2 on PC2. Similarly, the small α-helix made up of residues Gln147 to Lys151 was not observed in the conformations in the third-lowest energy basin centered around value zero on both PCs. However, the small β-sheet originating from the residues Val109 to Ser111 was found in the representative lowest energy conformation from this energy basin. The fourth lowest energy basin occupying 1.2 to 1.9 on PC1 and −1.2 to −1.9 on PC2 showed a unique helix region in the residues ranging from Gln147 to Lys151, while the small α-helix and a small β-sheet was absent in the representative lowest energy conformation. In the case of wild-type PON1, there were three lowest energy basins. The energy basin centered around −1 on PC1 and zero on PC2 had the small α-helix in the residues Gln147 to Lys151 in addition to the other two distinct α-helices (Figure 11). The largest energy basin showed two distinct conformations differing in the α-helices either in the residues between Cys42 and Val45 and the residues Gln147 to Lys151. The third lowest energy basin, relatively larger in size compared to the first energy basin, showed no distinct α-helices in the above-mentioned residues.

#### 3.3.3. Residue Contact Analysis

The effect of mutations was determined with contact frequency analysis in the case of mutated and wild-type PON1 with the tool mdciao using the cut-off distance of 4 Å. The residue–residue contact frequency analysis for Leu55Met suggested that the mutation Leu55Met resulted in variable close contacts between neighboring residues of Met55 with Phe64, Leu342, Val333, Gly344, Ile65, Ser335, Ile117, Ile343, Ala334, and Ala350 (Figure S1). The residues Phe64, Leu342, Val333, Gly344, Ile65, and Ser335 had frequency of more than 50% with Met55. Meanwhile, the Leu55 in Gln192Arg and wild-type PON1 had significantly close contacts with frequency of more than 50% with residues Phe64, Val333, Leu342, and Ser335. It was found that Met55 resulted in unique contacts with the residue Gly344 with more than 50% contact frequency, which otherwise was absent for Leu55 variants. The mutated Arg192 in Glu192Arg PON1 showed close contacts with His186, Phe186, and Leu187 with contact frequency of more than 50% and with residues Asp183, Asn166, Pro165, and Asn182 with less than 25% contact frequency. Residue Gln192 in leu55Met and wild-type variant showed close contacts with the same resides with more than 50% contact frequency, except Pro72 in the case of wild type. Further, the effect of Met55 mutation in Leu55Met is clearly evident in the contact frequency plot (Figure S2), where it showed significant contacts with residues Leu342, gly344, and Ile343 compared to Gln192Arg and wild-type variants. However, these contact frequencies were cut-off-distance-dependent where the cut-off distance of 4 Å was used. The cut-off independent frequency distribution analysis showed that Met55 had significant influence on contacts with Ala334, Ala350, and Ile353 residues, and such contacts are not seen in the Gln192Arg and wild-type variants. The Arg192 variant in Gln192Arg PON1 showed similar contact frequencies with residues Phe186, His184, and Leu187 compared to Leu55Met and wild-type variants (Figure S3). However, this Gln192Arg showed contacts with minor frequency below 25% with residues Asp183 and Asn166, which was absent in Leu55Met and wild-type variants. The distance-independent contact frequency distribution showed that the Gln192Arg variant has significant influence on residues Asn166 and Asn182. 

#### 3.3.4. Cross-Correlation Matrix of PON1

The DCCM analysis showed a strong negative or anti-correlation in the residues ranging from 50 to 75 and 160 to 180 in the Leu55Met PON1 variant (Figure S4). Further, the residues in the range 250 to 310 showed strong positive correlations. The Gln192Arg PON1 showed weaker positive and negative correlations in the residues in a similar range. In the case of wild-type PON1, a few of the residues in the range 160 to 180 showed strong negative correlations with the residues in the range 50 to 100.

### 3.4. Molecular Docking of Endogenous and Exogenous Substrate

Considering the change in the dynamics of PON1 wild-type and variant structures, we tested the binding affinity of their endogenous and exogenous substrates, for which their endogenous natural lactones (n = 4) and endogenous substrates (n = 6) were collected and docked against the wild-type and variant structures (Figures S5 and S6). The binding affinities of PON1 wild-type, Leu55Met, and Gln192Arg variant with endogenous natural lactones and synthetic endogenous substrates were shown in Table 2. Differences in the binding affinities were observed for the analyzed protein structures with the bound substrate, indicating conformational changes at the binding sites. 

## 4. Discussion

Oxidative stress is one of the primary events that contribute to a wide range of diseases [10,11,12,13,14,15]. Several endogenous antioxidant enzymes protect and maintain normal physiological function by combating oxidative stress [55]. PON1 is a well-known antioxidant protein whose activity is reported to be altered in a number of pathological conditions [56,57]. The decreased activity in diseases appears to be reciprocal, such that the disease significantly reduces PON1 activity or the genetic variant in PON1 may play a significant role in its activity that contributes to disease development. Several studies indicate that the genetic variants Leu55Met and Gln192Arg of PON1 contribute to a decrease in its activity across diseases [28,29,30,31,32]. However, the characteristic and specific structural consequences of these variants that cause functional changes are largely unknown. In our study, we used a series of computational methods to evaluate the effect of Leu55Met and Gln192Arg variants on the PON1 structure and the associated conformational changes at the substrate binding sites of its endogenous and exogenous compounds. Our implemented computational methods are interdependent. (1) Finding the most widely reported variants in PON1 has established our study’s importance across a variety of populations. (2) Generating the wild type, Leu55Met, and Gln192Arg of the PON1 structure helps to assess their structural differences. (3) The stability assessment tools demonstrated the consequences of these variants on structural stability. (4) With a basic understanding of structure stability, deeper insights were demonstrated through molecular dynamics simulation and essential dynamics investigation. (5) Finally, the molecular docking revealed the changes in the binding affinities of the endogenous and exogenous substrates that occurred due to variants that cause conformational changes at the substrate binding site of PON1 protein.

Firstly, we selected the template (PDB: 6H0A) to generate the PON1 wild type through homology modelling. In comparison to other templates, the chosen 6H0A template demonstrated superior quality in terms of QMEAN score, sequence identity, coverage, oligo state, and QMEANDisCo global scores, and structure resolutions that are well-suited for the generation of structure. Using the 6H0A, the wild-type structure was generated with SWISS-MODEL and validated using ProSa, QMEAN, PROCHECK, VERIFY-3D, and ERRAT tools. These validations served to affirm that the model produced was of sufficient quality to be utilized for subsequent analysis [34]. Using the validated wild-type PON1 structure, the variant structures were generated by substituting the appropriate amino acid (Gln192Arg and Leu55Met). Seven distinct protein stability assessment tools were utilized to evaluate the stability of the variant structures. These tools use distinct algorithms to predict the stability of proteins. Interestingly, most of these tools predict that both Leu55Met and Gln192Arg exhibit destabilizing effects to the protein structure despite the fact that these tools utilize different algorithms to predict the structural stability. Conversely, the stability-based DynaMut tool utilizes sampling conformations and the vibrational entropy alterations method for prediction. Nevertheless, the majority of the tools detected that the variants (Leu55Met and Gln192Arg) influence structure destabilization, which potentially leads to functional consequences, such as protein misfolding [58] and degradation [59], leading to loss/change in its function [60].

Based on a foundational comprehension of the alterations in the structural stability of PON1variants, further insights were revealed via implementing MD simulation and investigation of essential dynamics. PON1 wild type and variants showed distinct patterns in RMSD trajectories during MD simulation. Leu55Met exhibited higher deviations from the wild type, indicating its relative instability. In contrast, the Gln192Arg variant displayed lower RMSD value, indicating greater stability and rigidity compared to the wild type. Further, the RMSF analysis revealed that the Leu55Met variant had higher fluctuations, especially at amino acid 77PHE, in comparison to the wild type. Similarly, the Gln192Arg variant exhibited increased RMSF values at specific amino acid positions, although some regions overlapped with the wild type. Furthermore, the radius of gyration (Rg) of Leu55Met and Gln192Arg variant showed differences in trajectories during the simulation period compared to wild-type. However, the average Rg values of Leu55Met and Gln192Arg were similar to that of wild-type protein. Similarly, the solvent-accessible surface area (SASA) analysis showed that the Leu55Met and Gln192Arg variant showed relatively equal SASA average value to the wild type. Meanwhile, the average number of hydrogen bonds (HB) in the Leu55Met variant was less compared to the Gln192Arg and wild type, suggesting fewer interactions with the protein. Altogether, the dynamics results indicate that the PON1 with Leu55Met and variants differs significantly from the Gln192Arg and wild type. Particularly, the RMSD, RMSF, and H-bond results showed that PON1 with Leu55Met attains high flexibility and minimal flexibility with Gln192Arg when compared to the wild type. Also, increased HB was observed in the PON1 Gln192Arg and wild type compared to the Leu55Met protein, which might lead to loss in rigidity and instability in PON1 structure. Further, the essential dynamics analysis of PC1 and PC2 demonstrated that the Leu55Met (Figure 8) resulted in fewer meta-stable conformations with low-energy basin than the Gln192Arg (Figure 10) and wild type (Figure 11). Also, the energy barrier imposed seems to be in the transition of a few small secondary structural changes, such as α-helices made of Gly288 to Asp295 and Gln147 to Lys151 (Figure 9). The Gln192Arg variant has a relatively large number of meta-stable conformations, differing in a few of the smaller secondary structural aspects, including an α-helix made up of Val109 to Ser11 and a β-sheet made up of Cys42 to Val45 (Figure 10). The rest of the structure of the Gln192Arg variant is quite stable, as evident from the trajectories such as RMSD, RMSF, Rg, SASA, and HB. The wild-type PON1 also showed a relatively large number of low-energy meta-stable conformations, signifying its better stability compared to Leu55Met (Figure 11). The contact frequency analysis further confirms the effect of the Met55 mutation, leading to substantial contacts in the vicinity of this mutation compared to Gln192Arg and wild-type variants. The Met55 mutation leads to exclusive long-distance contacts with residues Ala334, Ala350, and Ile343, which might have resulted in the destabilization of this variant. On the other hand, the Gln192Arg variant showed long-distance contacts with Asn166 and Asn182 residues exclusively. However, the effect of the long-distance contact with residue Asn182 was very negligible. Compared to the Leu55Met variant, the Gln192Arg variant showed stable contacts with residues in the vicinity of this variant. The DCCM analysis further confirms the strong anti-correlating motions in Leu55Met in the residues ranging from 50 to 70 and residues 160 to 180. Meanwhile, such anti-correlating motions are fewer in Gln192Arg and wild-type PON1. Overall, the results of the essential dynamics supported the initial stability analysis and molecular dynamics trajectories that establish structure flexibility caused by Leu55Met substitution in PON1. This substitution may conceivably result in functional ramifications, including loss or alteration of the PON1 function.

Considerable change in the variants’ structure may affect the substrates binding capability of PON1. It is very important to recognize that the activities of PON1 that have been reported provide protection against diseases [7,8,9]. Particularly, the PON1 hydrolyze exogenous and endogenous substrates provide protection against substrate-induced toxicity [61,62]. Thereby, using a docking technique, we evaluated the impact of PON1 variant structures on the strength of the interaction with its exogenous and endogenous substrates. The results revealed a difference in the binding affinity between the wild-type and variant forms of PON1. In particular, the binding affinities of dihydrocoumarin, 2-hydroxy-gamma-butyrolactone, alpha-angelica lactone, arachidonic acid, and dihydropyran varied significantly between the wild-type and variant forms. Changes in the affinity may influence the metabolic turnover, which subsequently alter, the cellular process [63]. Interestingly, arachidonic acid is one of the analyzed substrates that showed notable changes in binding affinity with the Leu55Met variant (Table 2). Arachidonic acid plays a key role in cardiovascular disease, cancer, and other inflammatory conditions [64]. Particularly, arachidonic acid is implicated in the formation and progression of CVD [64,65] and is transformed into prostaglandin E2, which is involved in tumor extension [64,66] and also activates inflammation as an intercellular pro-inflammatory mediator [67]. Thereby, our findings underscore the significant role of variations in modifying the enzyme’s substrate interaction and its conformational landscape.

## 5. Conclusions

This study elucidates the effect of PON1 variants (Leu55Met and Gln192Arg) on the structural stability and binding efficacy of its exogenous and endogenous substrates through a series of computational approaches. Our structural stability analysis revealed that both Leu55Met and Gln192Arg have destabilizing effects that cause changes in protein dynamics. Particularly, the Leu55Met variant highly influences the PON1 structural stability more than wild-type and Gln192Arg during a 100 ns MD run through molecular and essential dynamics analysis. Further, substrate binding assessment in PON1 variants through molecular docking confirms changes in the binding efficacy of its exogenous and endogenous substrates, confirming a possible change in the PON1 function. Overall, our results indicate that Leu55Met variant may have a major impact on PON1 structure, which may change protein stability and perhaps disrupt cellular function, ultimately resulting in diseases linked to oxidative stress.

## Figures and Tables

**Table 1:** Protein stability analysis of Leu55Met, and Gln192Arg of PON1 structure.

Stability Assessment Tools	Leu55Met	Gln192Arg
Maestro Web	Destabilizing	Destabilizing
CUPSAT	Destabilizing	Destabilizing
DynaMut	Stabilizing	Stabilizing
I-Mutant	Destabilizing	Destabilizing
SDM2	Destabilizing	Destabilizing
PremPS	Destabilizing	Stabilizing

**Table 2:** Comparative least binding energies of natural and synthetic molecules with wild, Leu55Met, and Gln192Arg of PON1 structures.

Substrates	Wild Type	Leu55Met	Gln192Arg
**Endogenous Substrates (kcal/mol)**			
Dihydrocoumarin	−4.212	−5.137	−5.144
2-hydroxy-gamma-butyrolactone	−4.739	−4.077	−4.082
Alpha-angelica lactone	−4.361	−3.748	−3.665
Gamma-nonalactone	−3.35	−3.385	−3.537
**Exogenous Substrates (kcal/mol)**			
(2S,3R)-3-amino-2-hydroxy heptanoic acid	−5.461	−5.760	−5.380
Arachidonic acid	−3.453	−4.531	−3.949
4-Hydroxy docosahexaenoic acid	−4.081	−4.183	−4.288
Dihydropyran	−4.164	−3.781	−3.434
Mevalonic acid	−2.13	−2.516	−2.201
2-hydroxyvaleric acid	−2.914	−2.507	−2.324

## Data Availability

The data used to support the findings of this study are included within the article.

## Supplementary Materials

The following supporting information can be downloaded at: https://www.mdpi.com/article/10.3390/medicina59122060/s1, PON1 multiple Sequences; Figure S1. Contact frequency analysis; Figure S2. Contact frequency analysis for Leu55Met PON1 for Met55 mutation; Figure S3. Contact frequency analysis for Gln192Arg PON1; Figure S4. DCCM analysis; Figure S5. The PON1 wild-type docking with endogenous lactones; Figure S6. The interaction of PON1 wild-type with synthetic molecules.
