# Supplementary material for: Structural Characteristics of PON1 with Leu55Met and Gln192Arg Variants Influencing Oxidative-Stress-Related Diseases: An Integrated Molecular Modeling and Dynamics Study
Source: Medicina (Kaunas). 2023 Nov 22;59(12):2060. doi: 10.3390/medicina59122060 (PMC10744641; doi:10.3390/medicina59122060)
Supplement: Supplementary file 1 [file medicina-59-02060-s001.zip › medicina-2673962-supplementary.pdf]

PON1 multiple Sequences

PON1 Wild

MAKLIALTLLGMGLALFRNHQSSYQTRLNALREVQPVELPNCNLVKGIETGSEDLEILPNGLAFISSGLK
YPGIKSFNPNSPGKILLMDLNEEDPTVLELGITGSKFDVSSFNPHGISTFTDEDNAMYLLVVNHPDAKST
VELFKFQEEEKSLLHLKTIRHKLLPNLNDIVAVGPEHFYGTNDHYFLDPYLQSWEMYLGLAWSYVVYYS
PSEVRVVAEGFDFANGINISPDGKYVYIAELLAHKIHVYEKHANWTLTPLKSLDFNTLVDNISVDPETG
DLWVGCHPNGMKIFFYDSENPPASEVLRIQNILTEEPKVTQVYAENGTVLQGSTVASVYKGKLLIGTVF
HKALYCEL

L55M

MAKLIALTLLGMGLALFRNHQSSYQTRLNALREVQPVELPNCNLVKGIETGSED**M**EILPNGLAFISSGL
KYPGIKSFNPNSPGKILLMDLNEEDPTVLELGITGSKFDVSSFNPHGISTFTDEDNAMYLLVVNHPDAKS
TVELFKFQEEEKSLLHLKTIRHKLLPNLNDIVAVGPEHFYGTNDHYFLDPYLQSWEMYLGLAWSYVVY
YSPSEVRVVAEGFDFANGINISPDGKYVYIAELLAHKIHVYEKHANWTLTPLKSLDFNTLVDNISVDPET
GDLWVGCHPNGMKIFFYDSENPPASEVLRIQNILTEEPKVTQVYAENGTVLQGSTVASVYKGKLLIGTV
FHKALYCEL

Q192R

MAKLIALTLLGMGLALFRNHQSSYQTRLNALREVQPVELPNCNLVKGIETGSEDLEILPNGLAFISSGLK
YPGIKSFNPNSPGKILLMDLNEEDPTVLELGITGSKFDVSSFNPHGISTFTDEDNAMYLLVVNHPDAKST
VELFKFQEEEKSLLHLKTIRHKLLPNLNDIVAVGPEHFYGTNDHYFLDPYL**R**SWEMYLGLAWSYVVYYS
PSEVRVVAEGFDFANGINISPDGKYVYIAELLAHKIHVYEKHANWTLTPLKSLDFNTLVDNISVDPETG
DLWVGCHPNGMKIFFYDSENPPASEVLRIQNILTEEPKVTQVYAENGTVLQGSTVASVYKGKLLIGTVF
HKALYCEL

Figures S1. Contact frequency analysis. A) Leu55Met, B) Gln192Arg, and C) wild-type.

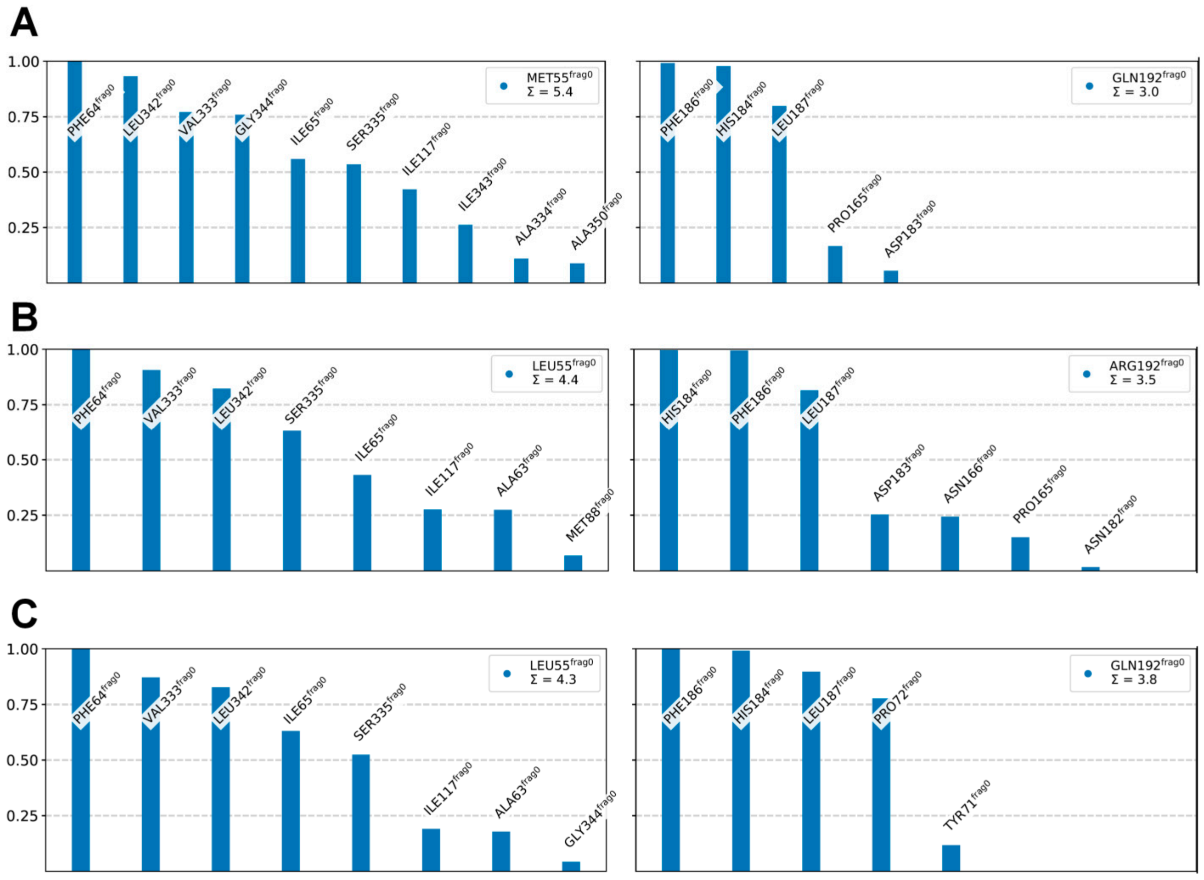

Figures S2. Contact frequency analysis for Leu55Met PON1 for Met55 mutation. The top panel plot shows the comparison in contact frequency between three variants of PON1, while the other panels show the distance independent contact frequency distribution comparison for Met55 mutation.

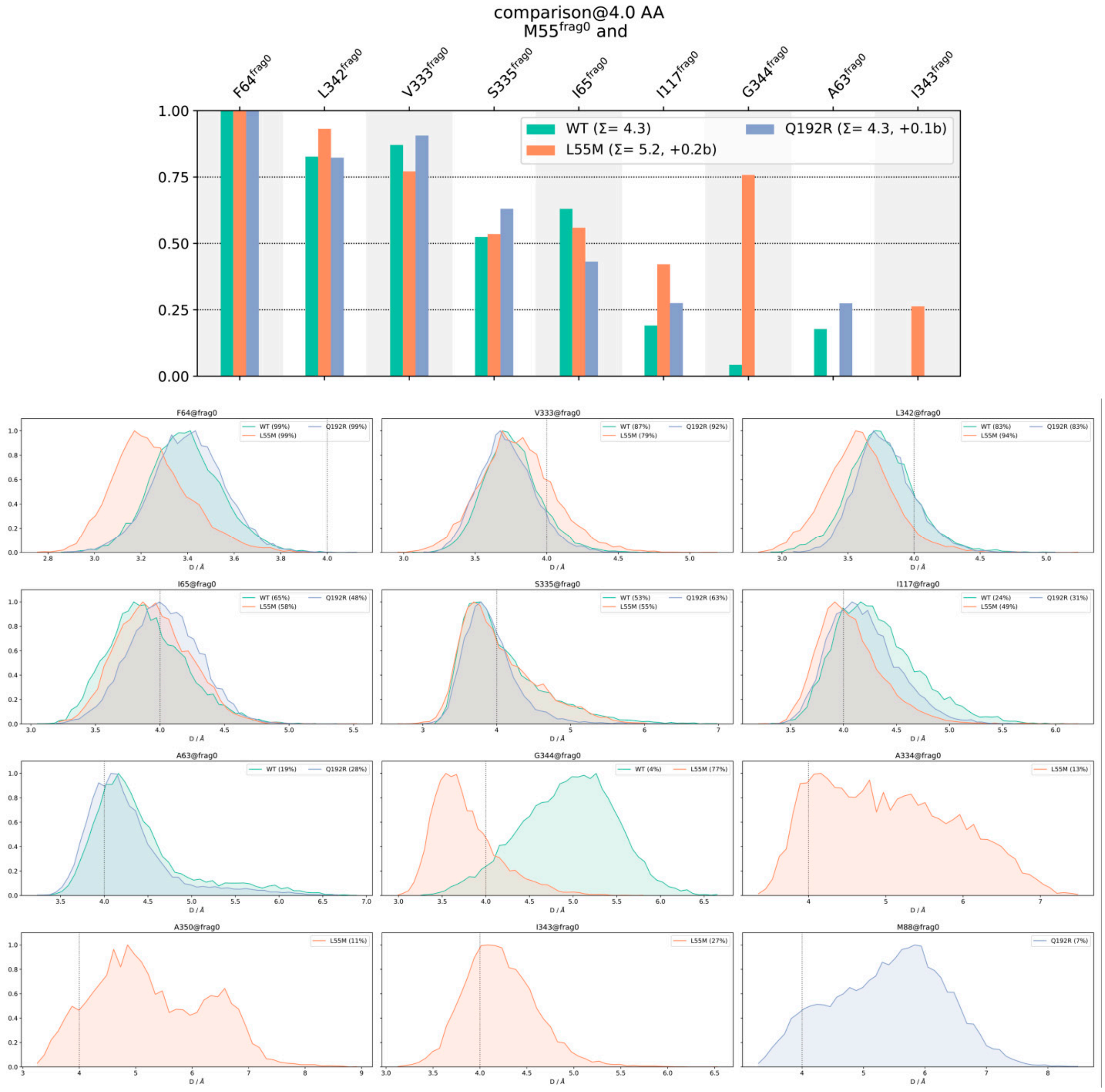

Figures S3. Contact frequency analysis for Gln192Arg PON1. The top panel plot shows the comparison in contact frequency between three variants of PON1 for Arg192 mutation, while the other panels show the distance independent contact frequency distribution comparison for Arg192 mutation.

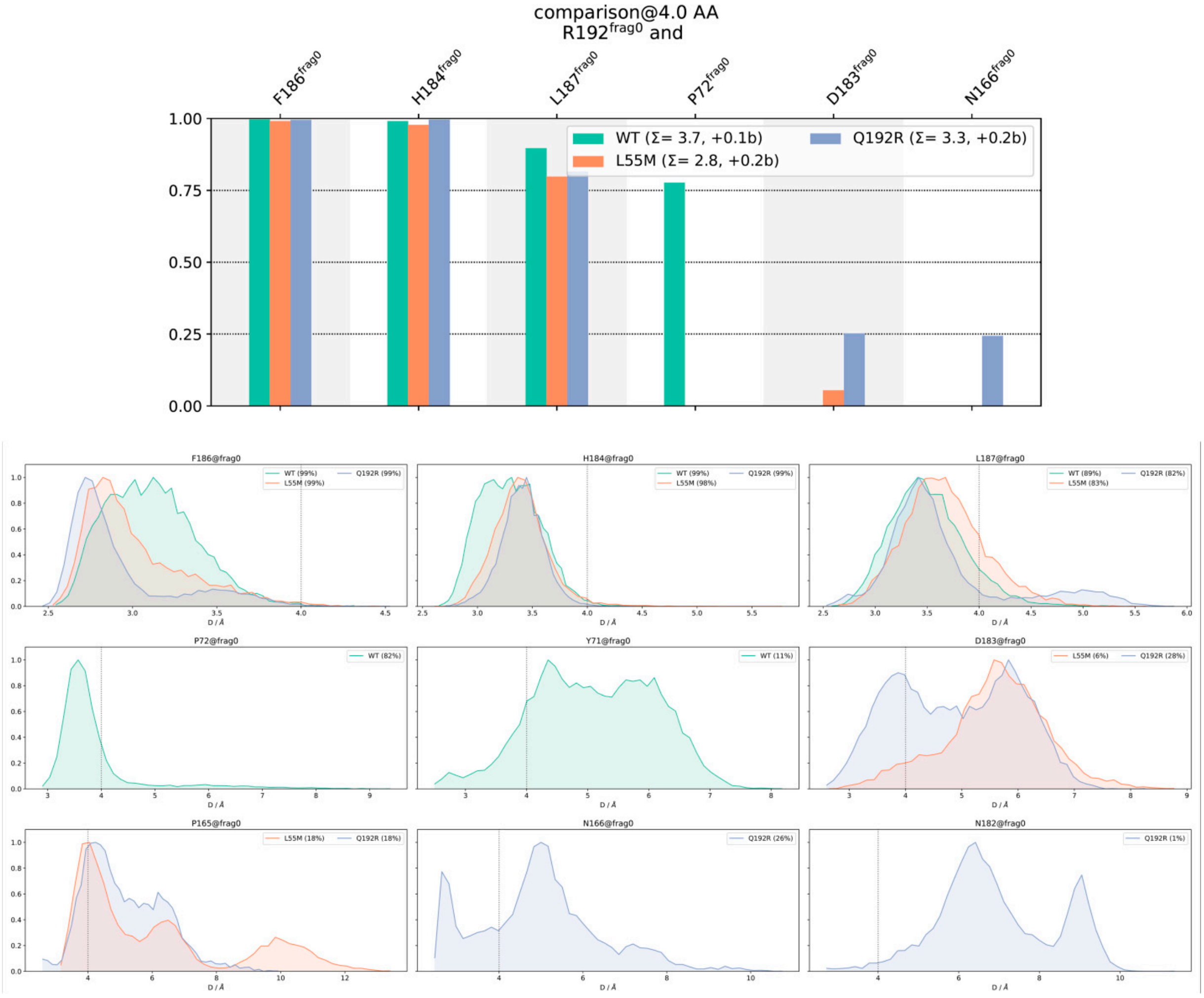

Figures S4. DCCM analysis. A) Leu55Met, B) Gln192Arg, and C) wild-type PON1

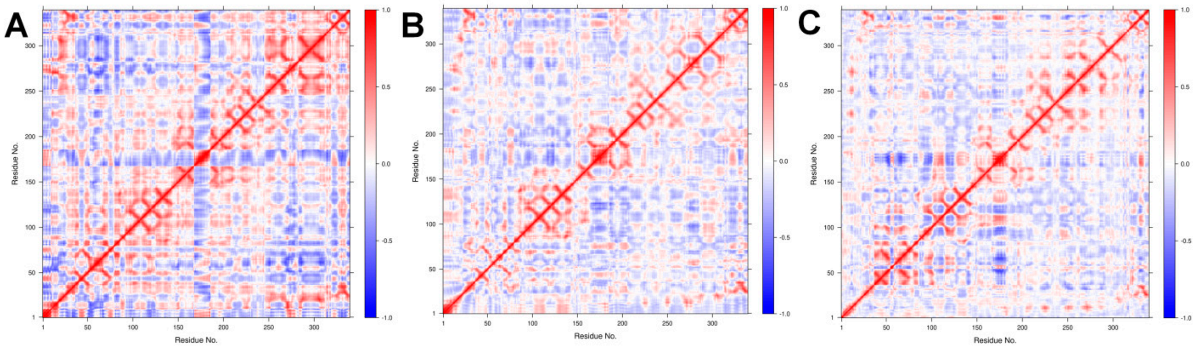

Figures S5. The PON1 wild-type docking with endogenous lactones: A) Dihydrocoumarin, B) 2-Hydroxy-gamma-butyrolactone C) Alpha-Angelica lactone, and D) Gamma-nonalactone. Leu55Met interacts with E) Dihydrocoumarin, F) 2-Hydroxy-gamma-butyrolactone G) Alpha-Angelica lactone, and H) Gam-ma-nonalactone. Gln192Arg interacts with I) Dihydrocoumarin, J) 2-Hydroxy-gamma-butyrolactone K) Alpha-Angelica lactone, and L) Gamma-non alac-tone.

A)

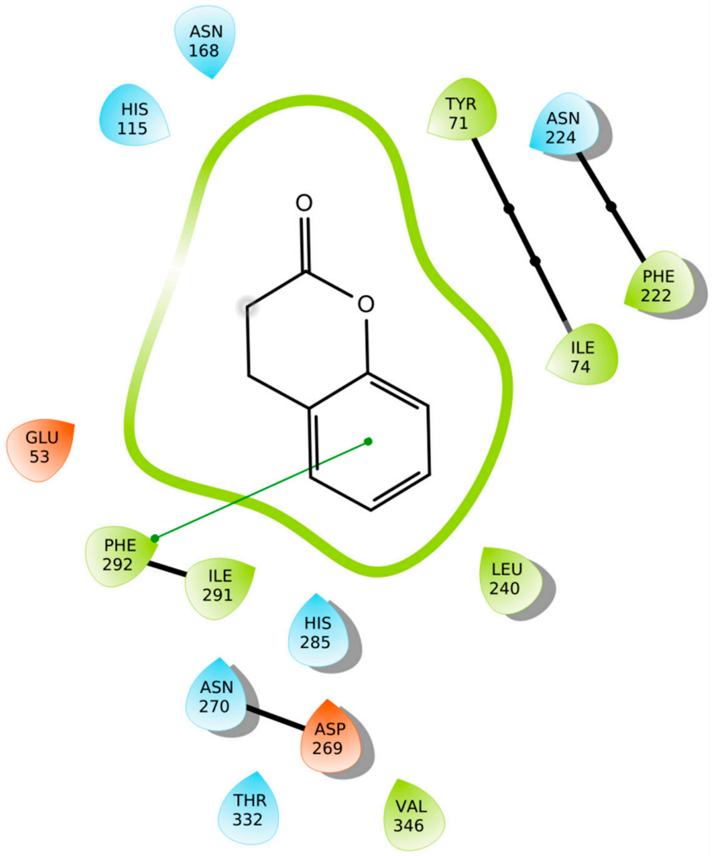

B)

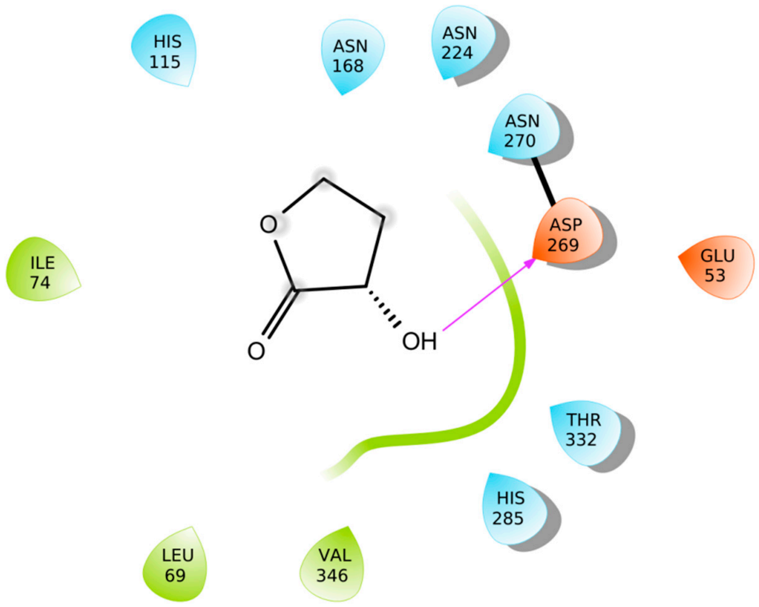

HIS 115
ASN 168
ASN 224
ASN 270
ASP 269
GLU 53
ILE 74
O
O
OH
THR 332
HIS 285
LEU 69
VAL 346

C)

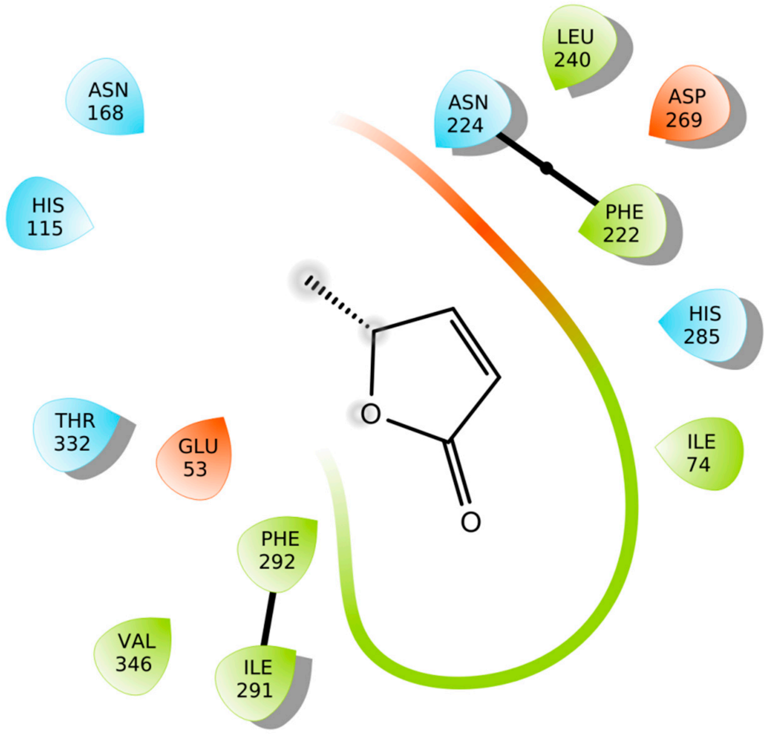

LEU 240
ASN 168
ASN 224
ASP 269
HIS 115
PHE 222
HIS 285
O
O
THR 332
GLU 53
ILE 74
PHE 292
VAL 346
ILE 291

D)

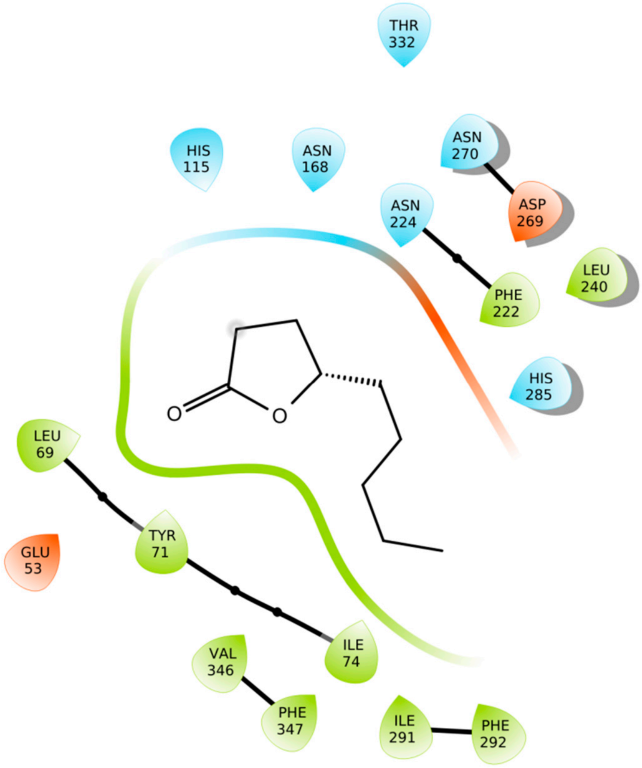
THR 332
HIS 115
ASN 168
ASN 270
ASP 269
ASN 224
LEU 240
PHE 222
HIS 285
O
O
LEU 69
GLU 53
TYR 71
ILE 74
VAL 346
PHE 347
ILE 291
PHE 292

E)

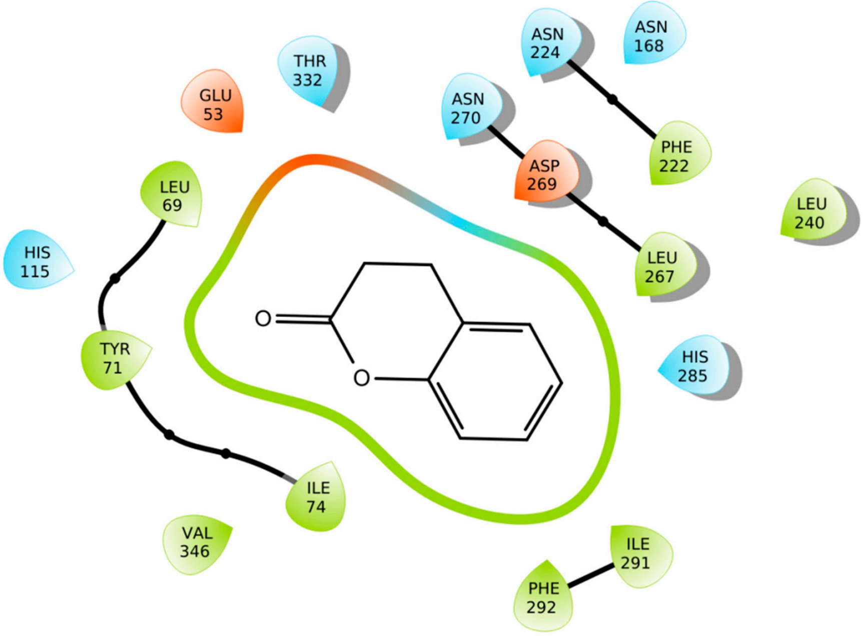
ASN 224
ASN 168
THR 332
GLU 53
ASN 270
PHE 222
ASP 269
LEU 69
LEU 240
HIS 115
LEU 267
O
O
TYR 71
HIS 285
ILE 74
VAL 346
ILE 291
PHE 292

F)

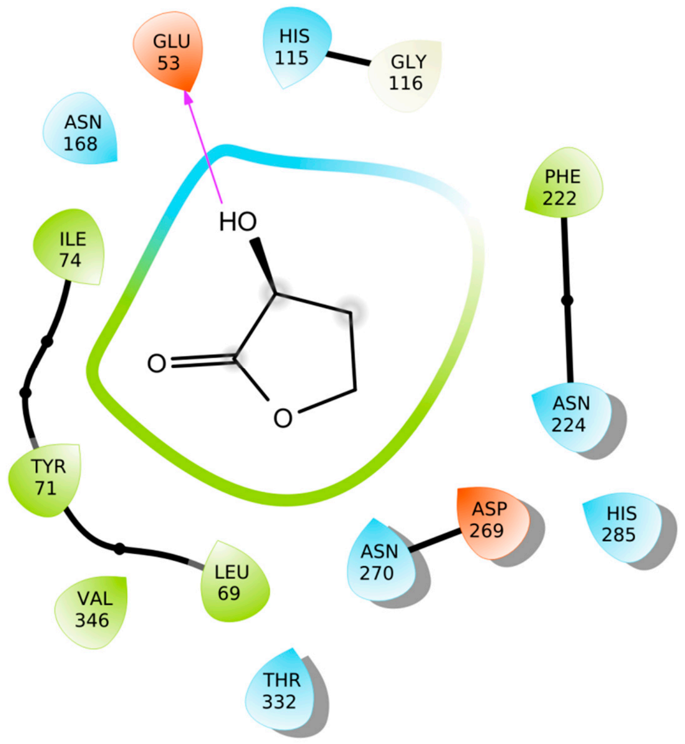
GLU
53
HIS
115
GLY
116
ASN
168
PHE
222
ILE
74
HO
O
O
ASN
224
TYR
71
ASP
269
HIS
285
ASN
270
LEU
69
VAL
346
THR
332

G)

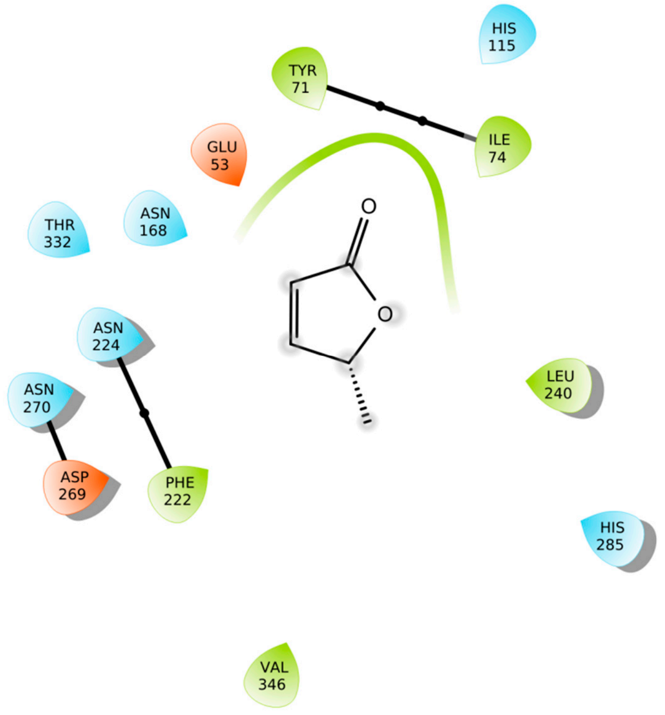
HIS
115
TYR
71
ILE
74
GLU
53
O
THR
332
ASN
168
O
ASN
224
LEU
240
ASN
270
ASP
269
PHE
222
HIS
285
VAL
346

H)

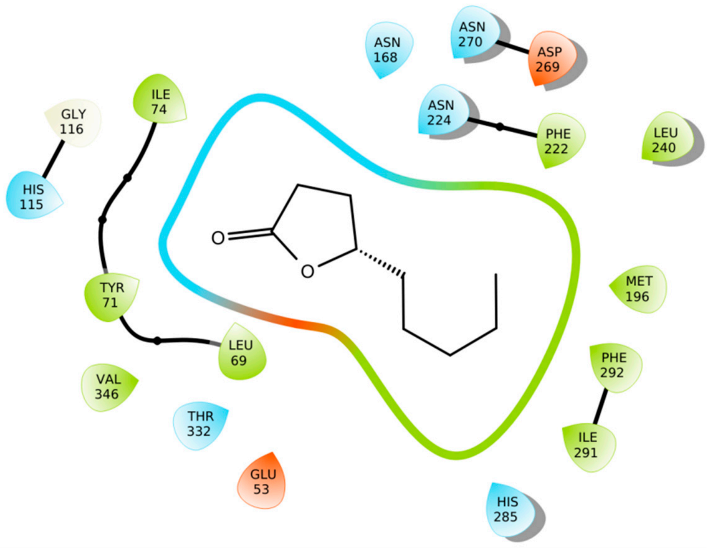
ASN 168
ASN 270
ASP 269
GLY 116
ILE 74
ASN 224
PHE 222
LEU 240
HIS 115
O
O
TYR 71
MET 196
LEU 69
PHE 292
VAL 346
THR 332
ILE 291
GLU 53
HIS 285

I)

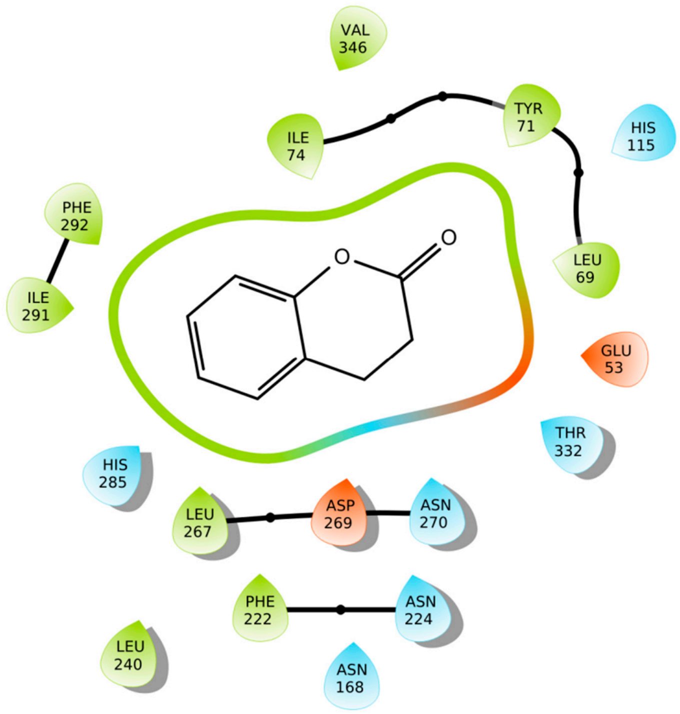
VAL 346
TYR 71
ILE 74
HIS 115
PHE 292
O
O
LEU 69
ILE 291
GLU 53
THR 332
HIS 285
LEU 267
ASP 269
ASN 270
PHE 222
ASN 224
LEU 240
ASN 168

J)

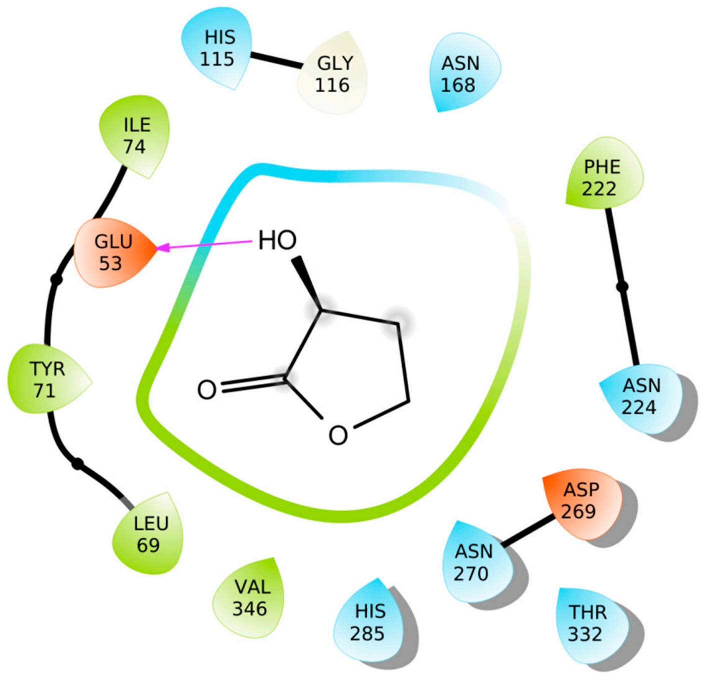
HIS
115
GLY
116
ASN
168
ILE
74
PHE
222
GLU
53
HO
TYR
71
O
O
ASN
224
ASP
269
LEU
69
ASN
270
VAL
346
HIS
285
THR
332

K)

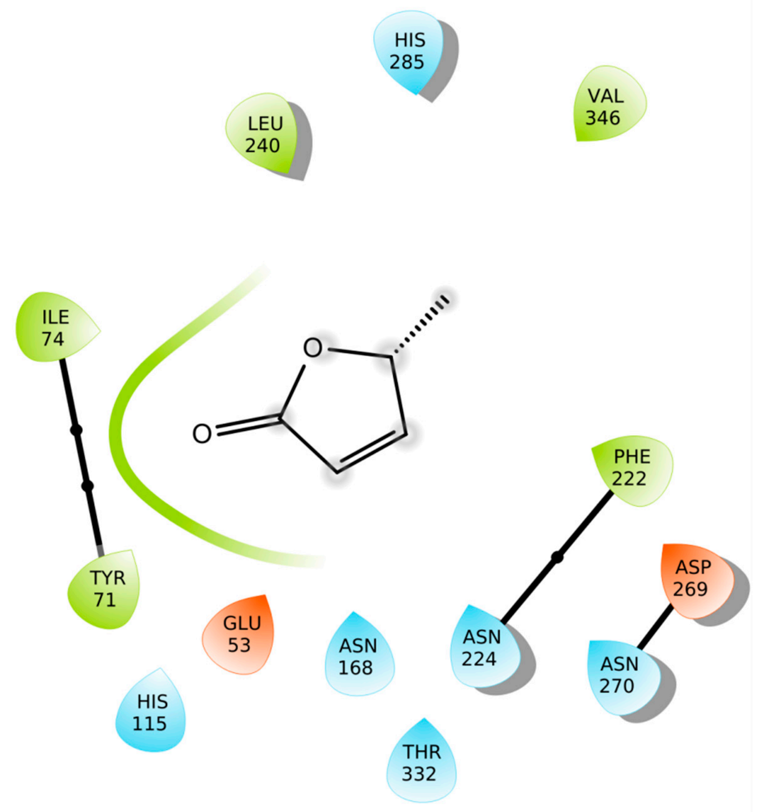
HIS
285
VAL
346
LEU
240
ILE
74
O
O
PHE
222
ASP
269
TYR
71
GLU
53
ASN
168
ASN
224
ASN
270
HIS
115
THR
332

L)

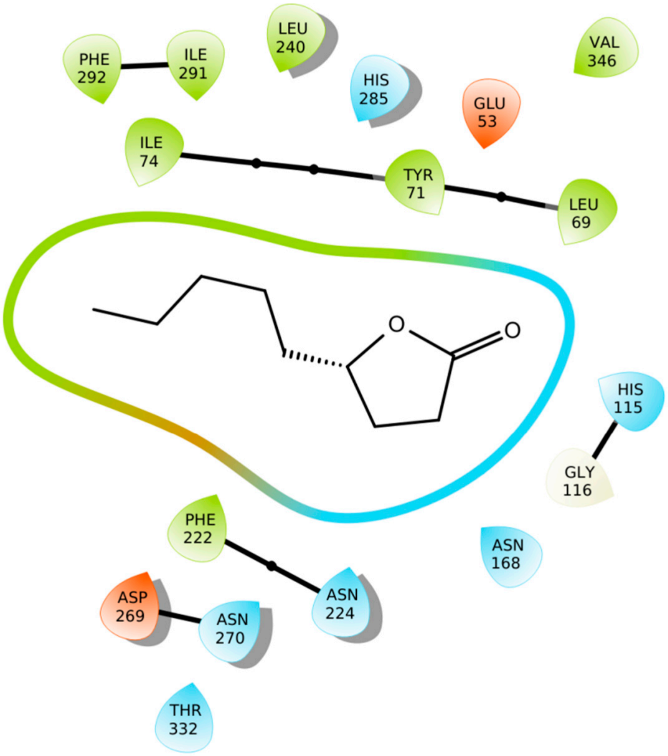
PHE
292
ILE
291
LEU
240
HIS
285
GLU
53
VAL
346
ILE
74
TYR
71
LEU
69
O
O
HIS
115
GLY
116
PHE
222
ASN
168
ASP
269
ASN
270
ASN
224
THR
332

Figures S6. The interaction of PON1 wild-type with synthetic molecules: A) (2S,3R)-3-amino-2-hydroxyheptonicacid, B) arachidonic acid, C) 4-hydroxy docosahexaenoic acid, D) dihydropyran, E) mevalonic acid,and F) 2-Hydroxyvaleric acid. Leu55Met docked with G) (2S,3R)-3-amino-2-hydroxyheptonicacid, H) arachidonic acid, I) 4-hydroxy docosahexaenoic acid, J) dihydropyran, K) mevalonic acid, and L) 2-Hydroxyvaleric acid. Gln192Arg docked with M)(2S,3R)-3-amino-2-hydroxyheptonicacid, N) arachidonic acid, O) 4-hydroxy docosahexaenoic acid, P) dihydropyran, Q) mevalonic acid, and R) 2-Hydroxyvaleric acid.

A)

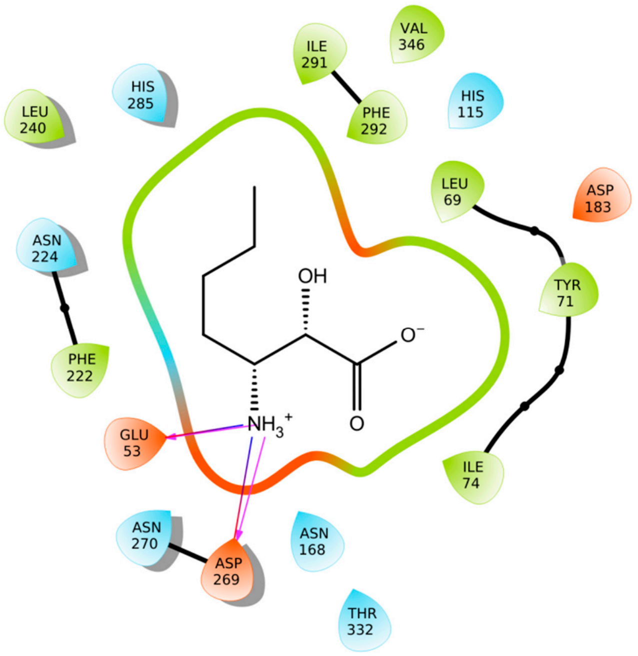

B)

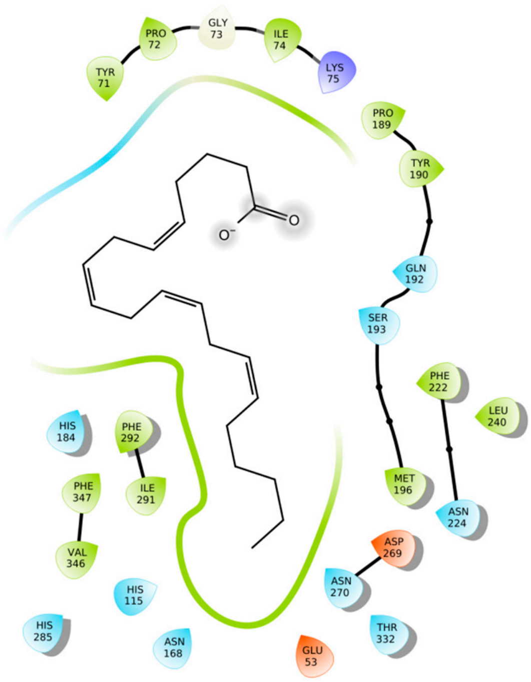

TYR 71
PRO 72
GLY 73
ILE 74
LYS 75
PRO 189
TYR 190
GLN 192
SER 193
PHE 222
LEU 240
MET 196
ASN 224
ASP 269
ASN 270
THR 332
GLU 53
ASN 168
HIS 285
HIS 115
VAL 346
PHE 347
ILE 291
PHE 292
HIS 184
O-
O

C)

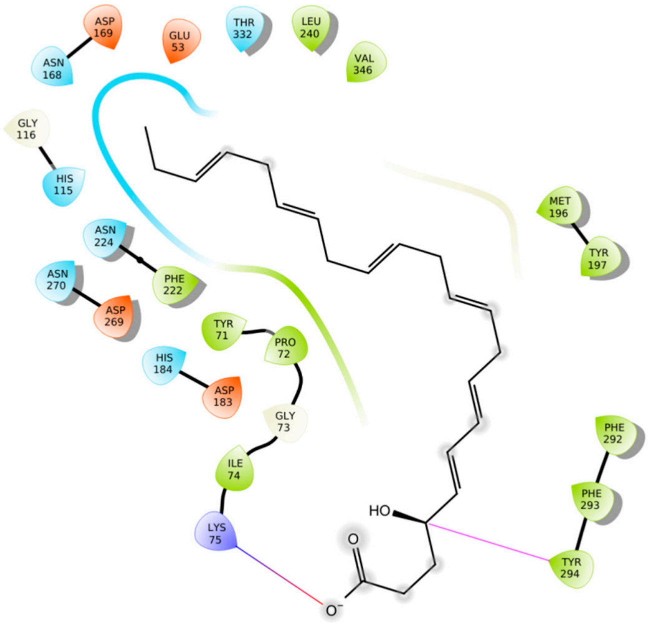

ASP 169
GLU 53
THR 332
LEU 240
VAL 346
ASN 168
GLY 116
HIS 115
ASN 224
PHE 222
ASN 270
ASP 269
TYR 71
PRO 72
HIS 184
ASP 183
GLY 73
ILE 74
LYS 75
MET 196
TYR 197
PHE 292
PHE 293
TYR 294
HO
O
O-

D)

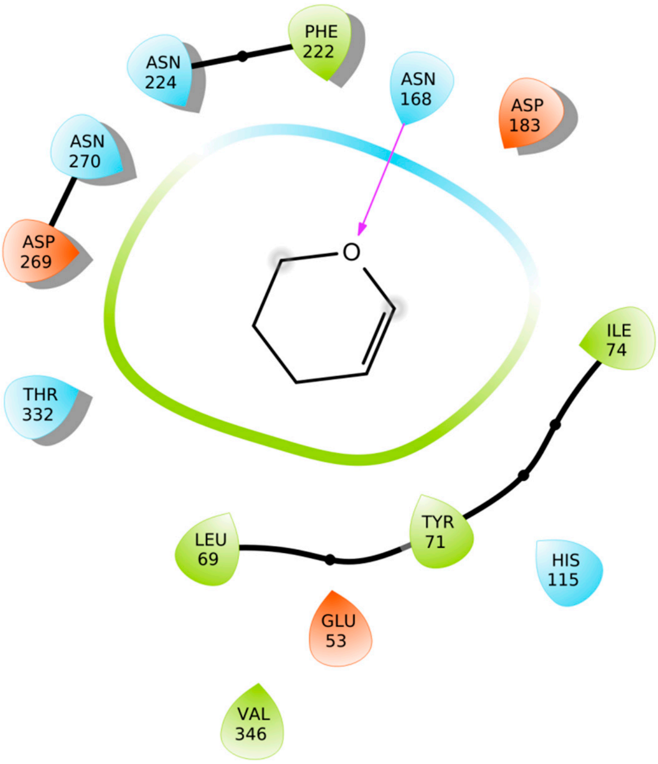
PHE
222
ASN
224
ASN
168
ASP
183
ASN
270
O
ASP
269
ILE
74
THR
332
LEU
69
TYR
71
HIS
115
GLU
53
VAL
346

E)

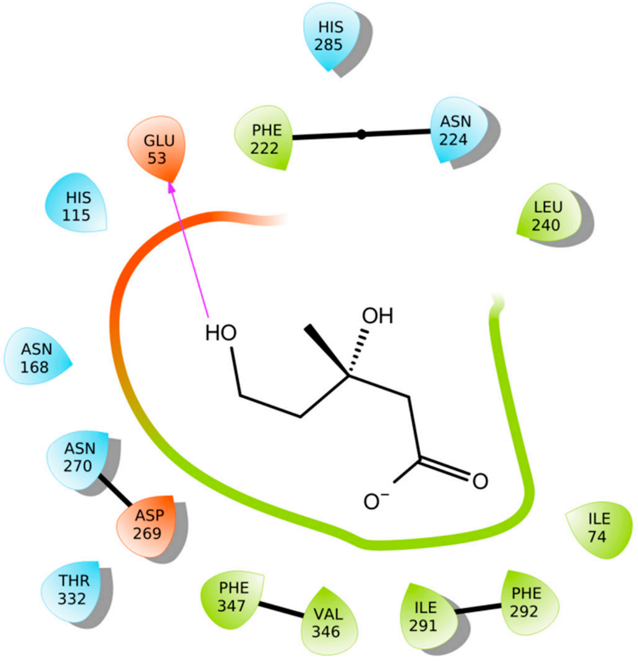
HIS
285
PHE
222
ASN
224
GLU
53
HIS
115
LEU
240
HO
OH
ASN
168
O
O⁻
ASN
270
ASP
269
ILE
74
THR
332
PHE
347
VAL
346
ILE
291
PHE
292

F)

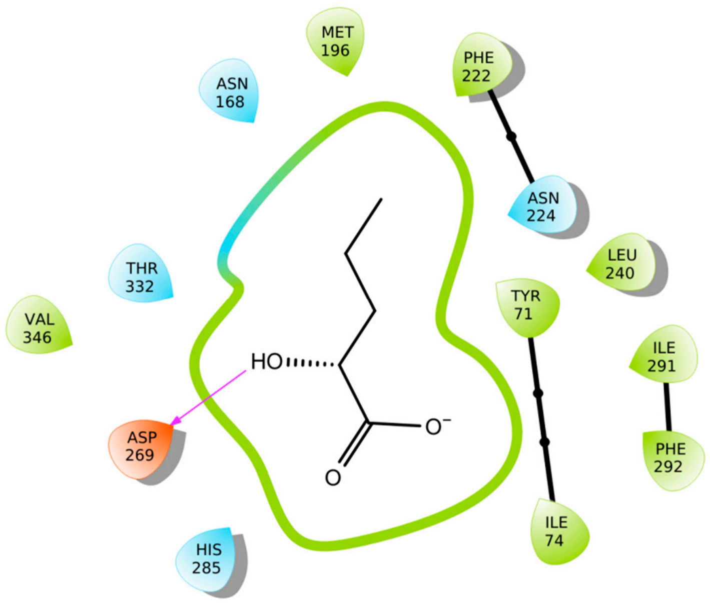
MET 196
ASN 168
PHE 222
ASN 224
LEU 240
THR 332
TYR 71
VAL 346
HO
ILE 291
ASP 269
O−
PHE 292
O
ILE 74
HIS 285

G)

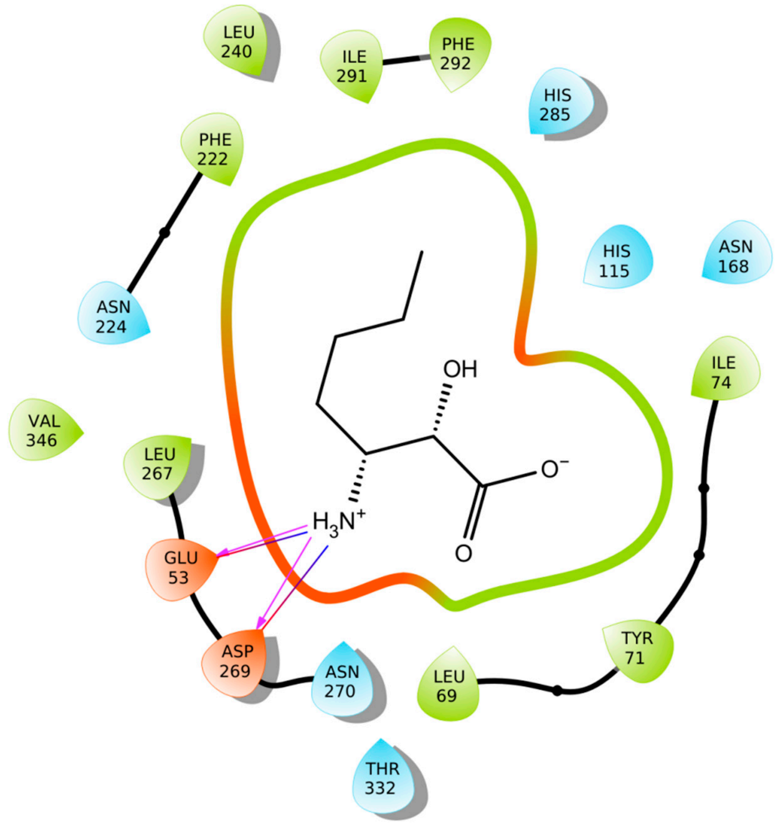
LEU 240
ILE 291
PHE 292
HIS 285
PHE 222
HIS 115
ASN 168
ASN 224
OH
ILE 74
VAL 346
LEU 267
O−
H3N+
GLU 53
O
ASP 269
ASN 270
LEU 69
TYR 71
THR 332

H)

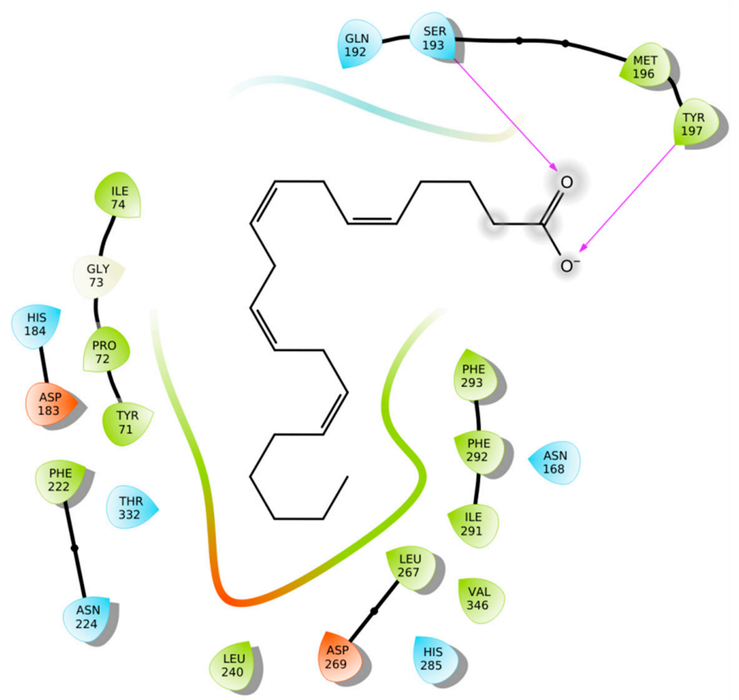

GLN 192
SER 193
MET 196
TYR 197
ILE 74
GLY 73
HIS 184
PRO 72
ASP 183
TYR 71
PHE 222
THR 332
ASN 224
LEU 240
ASP 269
LEU 267
HIS 285
VAL 346
PHE 293
PHE 292
ASN 168
ILE 291
O
O⁻

I)

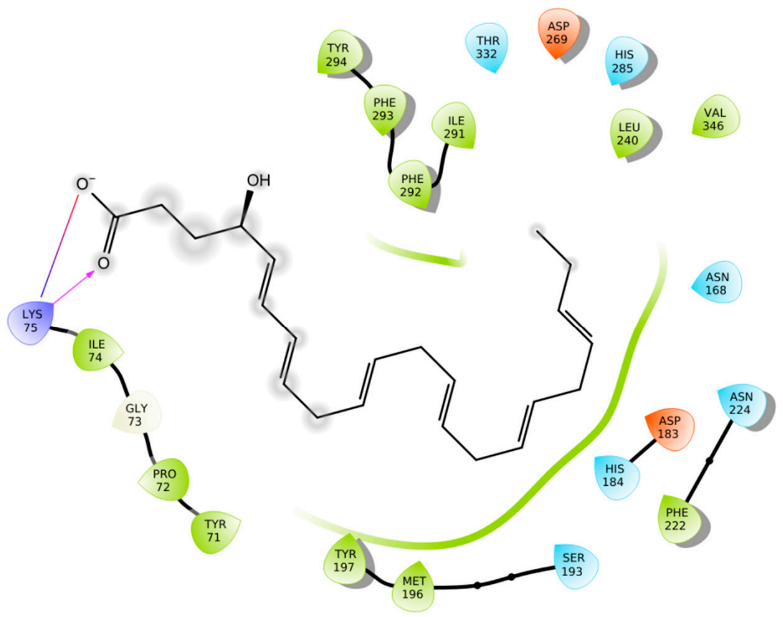

TYR 294
THR 332
ASP 269
HIS 285
PHE 293
ILE 291
LEU 240
VAL 346
PHE 292
OH
O⁻
O
LYS 75
ILE 74
GLY 73
PRO 72
TYR 71
ASN 168
ASP 183
ASN 224
HIS 184
PHE 222
TYR 197
MET 196
SER 193

J)

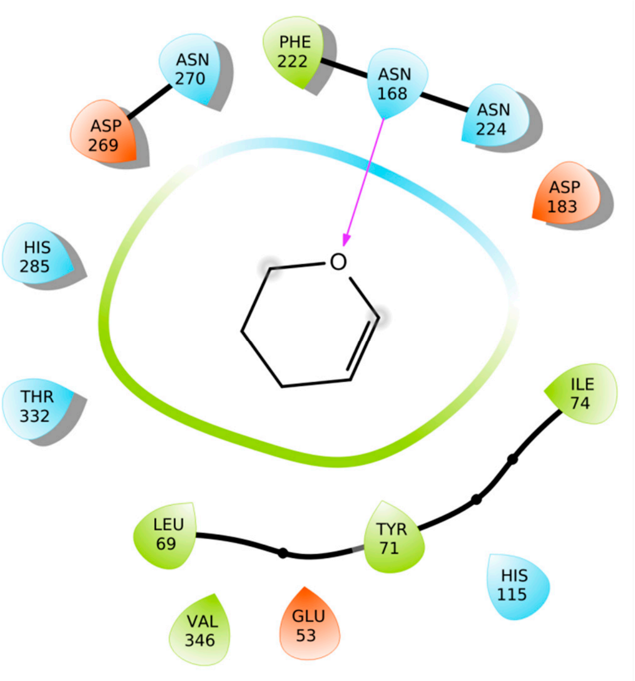
PHE 222
ASN 270
ASN 168
ASN 224
ASP 269
ASP 183
HIS 285
O
THR 332
ILE 74
LEU 69
TYR 71
HIS 115
VAL 346
GLU 53

K)

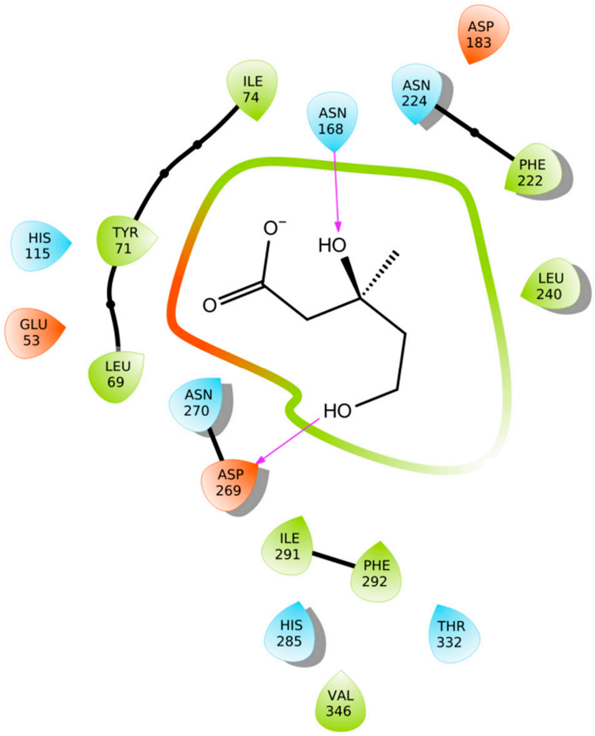
ASP 183
ILE 74
ASN 224
ASN 168
PHE 222
HIS 115
TYR 71
O⁻
HO
LEU 240
O
GLU 53
LEU 69
ASN 270
HO
ASP 269
ILE 291
PHE 292
HIS 285
THR 332
VAL 346

L)

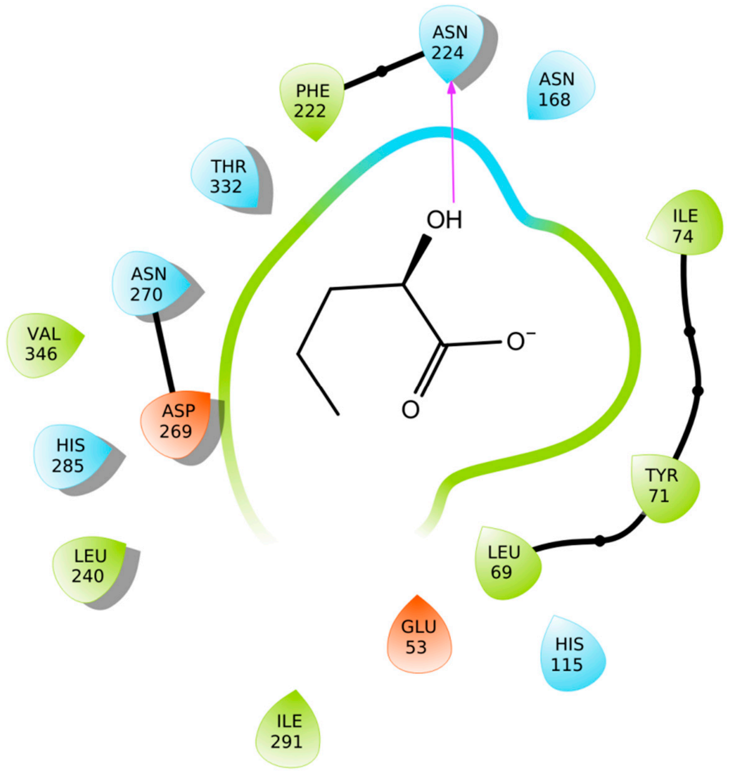
ASN 224
PHE 222
ASN 168
THR 332
OH
ILE 74
ASN 270
VAL 346
O⁻
ASP 269
O
HIS 285
TYR 71
LEU 240
LEU 69
GLU 53
HIS 115
ILE 291

M)

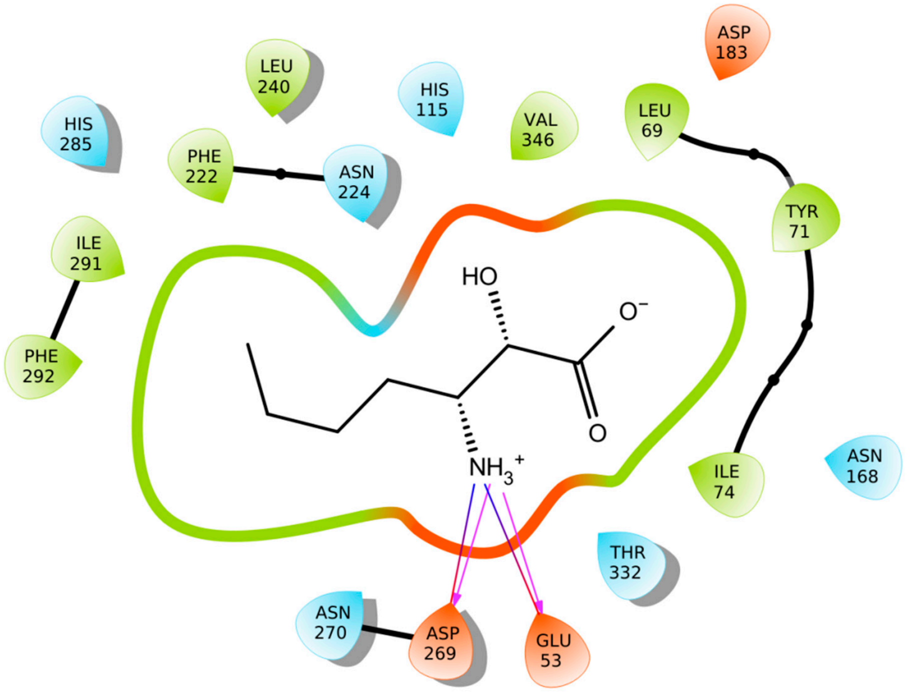
ASP 183
LEU 240
HIS 115
HIS 285
VAL 346
LEU 69
PHE 222
ASN 224
TYR 71
ILE 291
HO
O⁻
PHE 292
O
NH_3^+
ILE 74
ASN 168
THR 332
ASN 270
ASP 269
GLU 53

N)

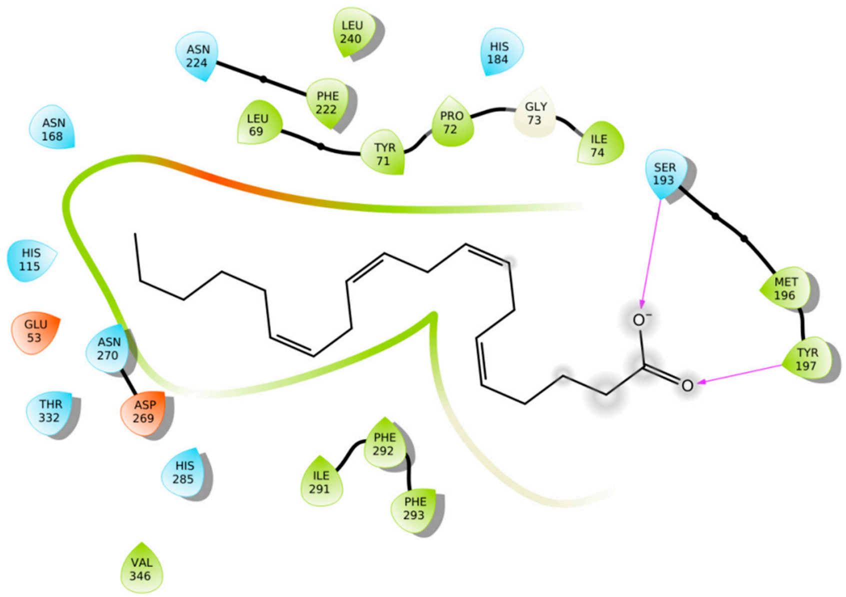

LEU 240
ASN 224
HIS 184
PHE 222
ASN 168
LEU 69
PRO 72
GLY 73
TYR 71
ILE 74
SER 193
HIS 115
MET 196
O⁻
GLU 53
ASN 270
TYR 197
O
THR 332
ASP 269
PHE 292
HIS 285
ILE 291
PHE 293
VAL 346

O)

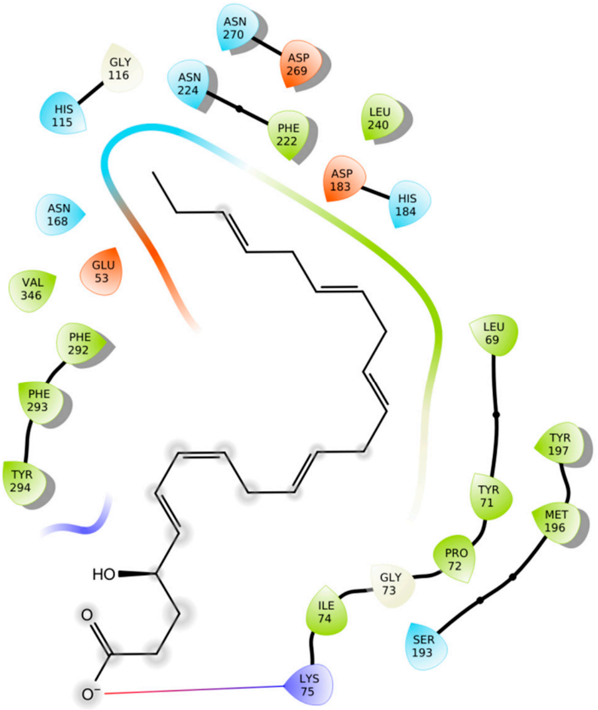

ASN 270
ASP 269
GLY 116
ASN 224
HIS 115
PHE 222
LEU 240
ASP 183
HIS 184
ASN 168
GLU 53
VAL 346
PHE 292
LEU 69
PHE 293
TYR 197
TYR 294
TYR 71
MET 196
HO
PRO 72
GLY 73
ILE 74
O
SER 193
LYS 75
O⁻

P)

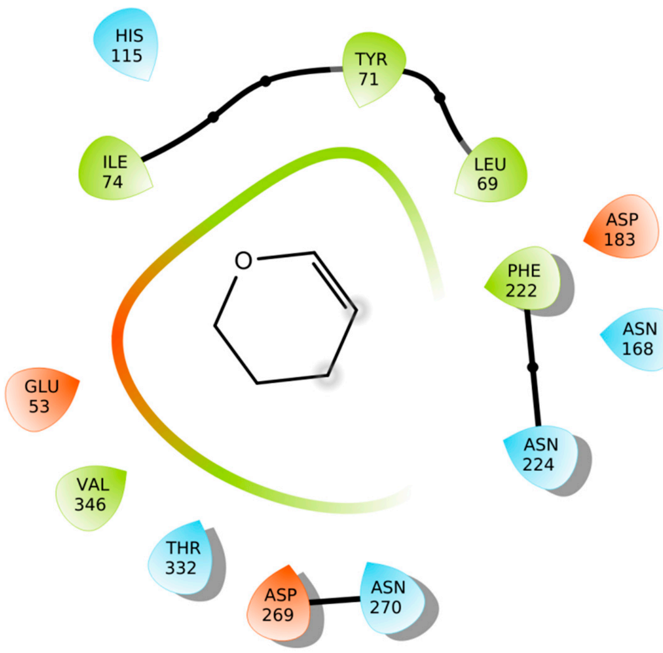
HIS
115
TYR
71
ILE
74
LEU
69
ASP
183
O
PHE
222
ASN
168
GLU
53
ASN
224
VAL
346
THR
332
ASP
269
ASN
270

Q)

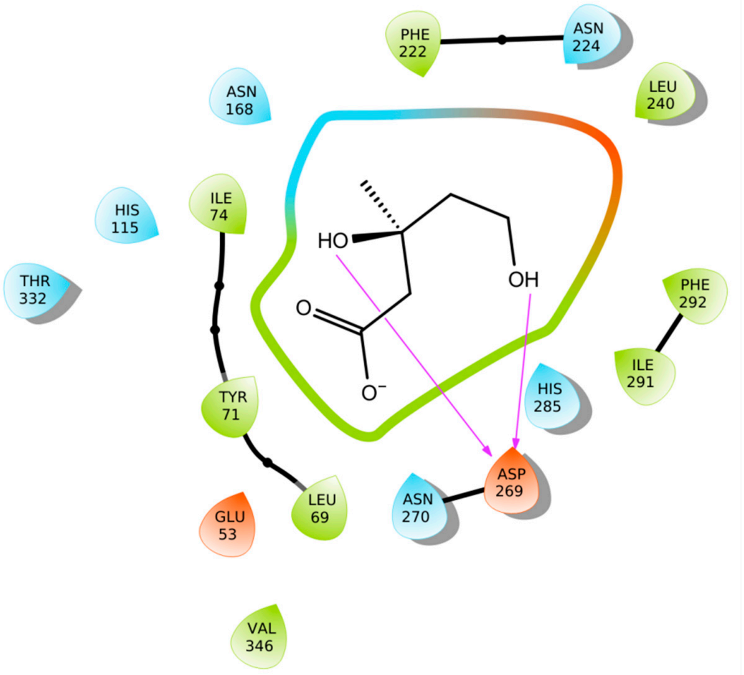
PHE
222
ASN
224
LEU
240
ASN
168
HIS
115
ILE
74
HO
OH
THR
332
O
O⁻
PHE
292
ILE
291
HIS
285
TYR
71
ASP
269
ASN
270
LEU
69
GLU
53
VAL
346

R)

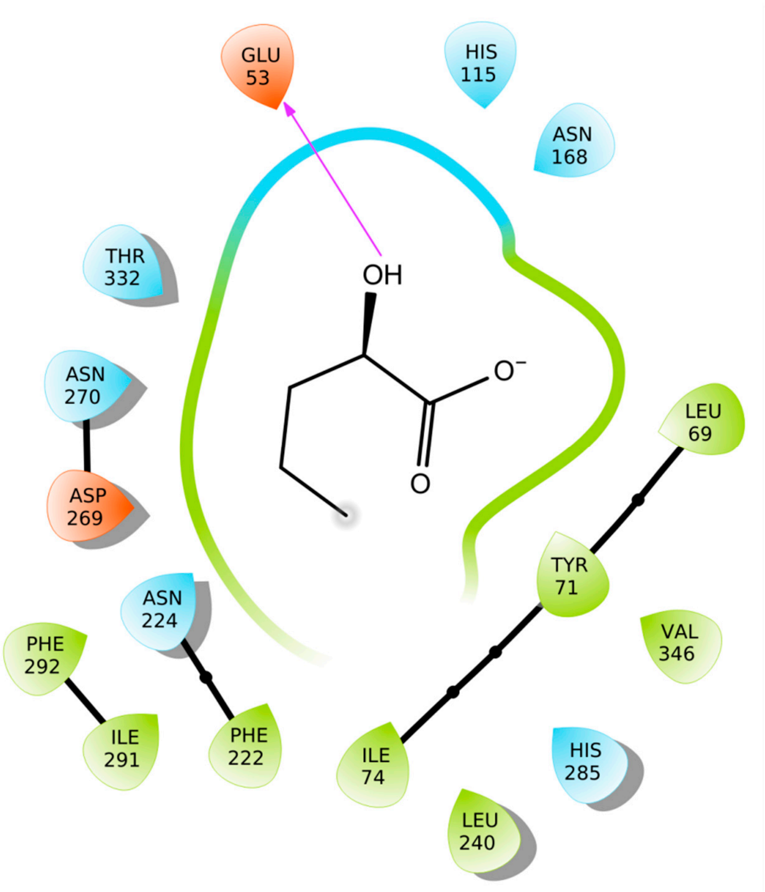

GLU 53
HIS 115
ASN 168
THR 332
OH
O⁻
O
ASN 270
ASP 269
LEU 69
TYR 71
ASN 224
VAL 346
PHE 292
ILE 291
PHE 222
ILE 74
HIS 285
LEU 240
